# BA.2.12.1, BA.4 and BA.5 escape antibodies elicited by Omicron infection

**DOI:** 10.1038/s41586-022-04980-y

**Published:** 2022-06-17

**Authors:** Yunlong Cao, Ayijiang Yisimayi, Fanchong Jian, Weiliang Song, Tianhe Xiao, Lei Wang, Shuo Du, Jing Wang, Qianqian Li, Xiaosu Chen, Yuanling Yu, Peng Wang, Zhiying Zhang, Pulan Liu, Ran An, Xiaohua Hao, Yao Wang, Jing Wang, Rui Feng, Haiyan Sun, Lijuan Zhao, Wen Zhang, Dong Zhao, Jiang Zheng, Lingling Yu, Can Li, Na Zhang, Rui Wang, Xiao Niu, Sijie Yang, Xuetao Song, Yangyang Chai, Ye Hu, Yansong Shi, Linlin Zheng, Zhiqiang Li, Qingqing Gu, Fei Shao, Weijin Huang, Ronghua Jin, Zhongyang Shen, Youchun Wang, Xiangxi Wang, Junyu Xiao, Xiaoliang Sunney Xie

**Affiliations:** 1 Biomedical Pioneering Innovation Center (BIOPIC), Peking University, Beijing, P. R. China; 2 Changping Laboratory, Beijing, P. R. China; 3 School of Life Sciences, Peking University, Beijing, P. R. China; 4 College of Chemistry and Molecular Engineering, Peking University, Beijing, P. R. China; 5 Joint Graduate Program of Peking–Tsinghua–NIBS, Academy for Advanced Interdisciplinary Studies, Peking University, Beijing, P. R. China; 6 CAS Key Laboratory of Infection and Immunity, National Laboratory of Macromolecules, Institute of Biophysics, Chinese Academy of Sciences, Beijing, P. R. China; 7 Division of HIV/AIDS and Sex-transmitted Virus Vaccines, Institute for Biological Product Control, National Institutes for Food and Drug Control (NIFDC), Beijing, P. R. China; 8 Institute for Immunology, College of Life Sciences, Nankai University, Tianjin, P. R. China; 9 Beijing Ditan Hospital, Capital Medical University, Beijing, P. R. China; 10 Peking–Tsinghua Center for Life Sciences, Peking University, Beijing, P. R. China; 11 Academy for Advanced Interdisciplinary Studies, Peking University, Beijing, P. R. China; 12 Organ Transplant Center, NHC Key Laboratory for Critical Care Medicine, Tianjin First Central Hospital, Nankai University, Tianjin, P. R. China

**Keywords:** Immunology, Antibodies, SARS-CoV-2

## Abstract

Severe acute respiratory syndrome coronavirus 2 (SARS-CoV-2) Omicron sublineages BA.2.12.1, BA.4 and BA.5 exhibit higher transmissibility than the BA.2 lineage^1^. The receptor binding and immune-evasion capability of these recently emerged variants require immediate investigation. Here, coupled with structural comparisons of the spike proteins, we show that BA.2.12.1, BA.4 and BA.5 (BA.4 and BA.5 are hereafter referred collectively to as BA.4/BA.5) exhibit similar binding affinities to BA.2 for the angiotensin-converting enzyme 2 (ACE2) receptor. Of note, BA.2.12.1 and BA.4/BA.5 display increased evasion of neutralizing antibodies compared with BA.2 against plasma from triple-vaccinated individuals or from individuals who developed a BA.1 infection after vaccination. To delineate the underlying antibody-evasion mechanism, we determined the escape mutation profiles^2^, epitope distribution^3^ and Omicron-neutralization efficiency of 1,640 neutralizing antibodies directed against the receptor-binding domain of the viral spike protein, including 614 antibodies isolated from people who had recovered from BA.1 infection. BA.1 infection after vaccination predominantly recalls humoral immune memory directed against ancestral (hereafter referred to as wild-type (WT)) SARS-CoV-2 spike protein. The resulting elicited antibodies could neutralize both WT SARS-CoV-2 and BA.1 and are enriched on epitopes on spike that do not bind ACE2. However, most of these cross-reactive neutralizing antibodies are evaded by spike mutants L452Q, L452R and F486V. BA.1 infection can also induce new clones of BA.1-specific antibodies that potently neutralize BA.1. Nevertheless, these neutralizing antibodies are largely evaded by BA.2 and BA.4/BA.5 owing to D405N and F486V mutations, and react weakly to pre-Omicron variants, exhibiting narrow neutralization breadths. The therapeutic neutralizing antibodies bebtelovimab^4^ and cilgavimab^5^ can effectively neutralize BA.2.12.1 and BA.4/BA.5, whereas the S371F, D405N and R408S mutations undermine most broadly sarbecovirus-neutralizing antibodies. Together, our results indicate that Omicron may evolve mutations to evade the humoral immunity elicited by BA.1 infection, suggesting that BA.1-derived vaccine boosters may not achieve broad-spectrum protection against new Omicron variants.

## Abstract (web summary)

Biochemical and structural studies of the interactions between antibodies and spike proteins from SARS-CoV-2 Omicron subvariants indicate how these variants have evolved to escape antibody-mediated neutralization.

## Main

**Extended Data Fig. 1:**
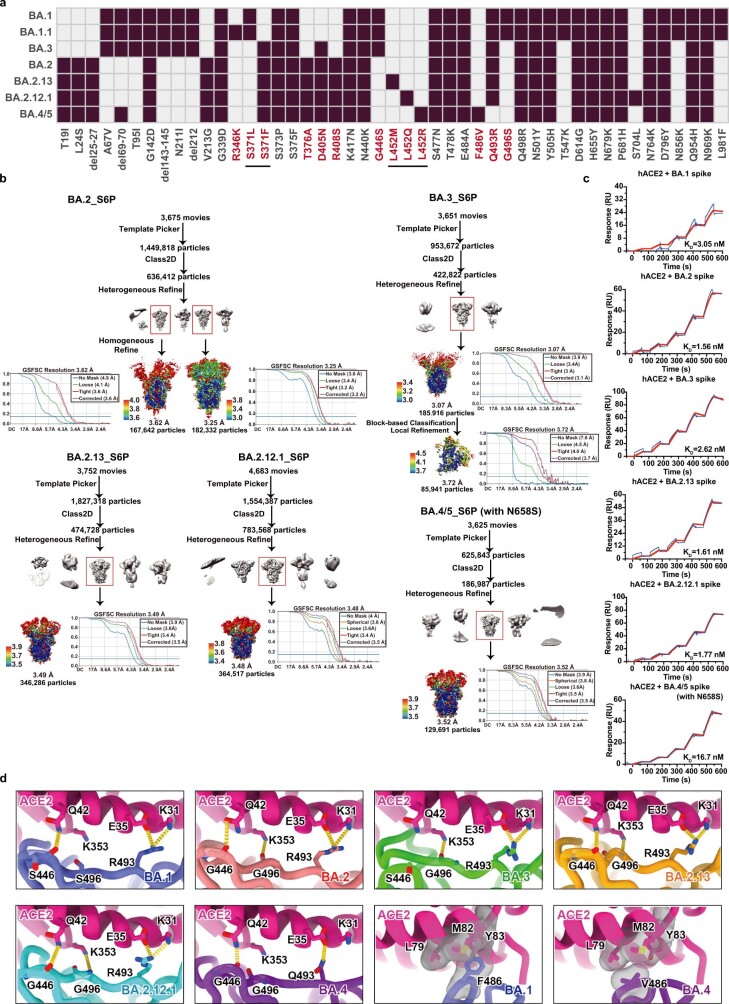
Structures and ACE2 binding of emerging Omicron subvariants spike glycoprotein. **a**, Mutations on the spike glycoprotein of SARS-CoV-2 Omicron subvariants. Residues that are not identical among Omicron subvariants are colored red. **b**, Workflow to generate cryo-EM structure of BA.2, BA.3, BA.2.13, BA.2.12.1, BA.4/5 spike glycoprotein trimer with S6P and R683A, R685A substitutions. **c**, Binding affinities of Omicron variants spike trimers to hACE2 measured by SPR. SPR analyses were conducted in biological duplicates. **d**, MD simulated interactions between hACE2 and RBD of Omicron variants. Structures of the RBD from Omicron variants and hACE2 are shown as ribbons.

The recent emergence and global spread of the SARS-CoV-2 variant Omicron (B.1.1.529) have posed a critical challenge to the efficacy of COVID-19 vaccines and neutralizing antibody (NAb) therapy^6–9^. Owing to multiple mutations to the spike protein, including in the receptor-binding domain (RBD) and N-terminal domain, Omicron BA.1 infection can result in substantial NAb evasion^3,10–13^. Omicron sublineage BA.2 has rapidly surged worldwide, out-competing BA.1. Compared with the RBD of BA.1, BA.2 contains three additional mutations, T376A, D405N and R408S, and lacks the BA.1 mutations G446S and G496S (Extended Data Fig. 1a). S371L on BA.1 is also substituted with S371F in BA.2. The Omicron variants that have emerged more recently contain similar RBD sequences to BA.2, with the addition of L452 and F486 substitutions—L452Q in BA.2.12.1, L452M in BA.2.13 and L452R/F486V in BA.4 and BA.5—and exhibit a transmission advantage over BA.2. There is an urgent and immediate need to investigate the receptor binding and immune-evasion capabilities of these new Omicron variants.

## Structural analyses of Omicron spike

**Fig. 1:**
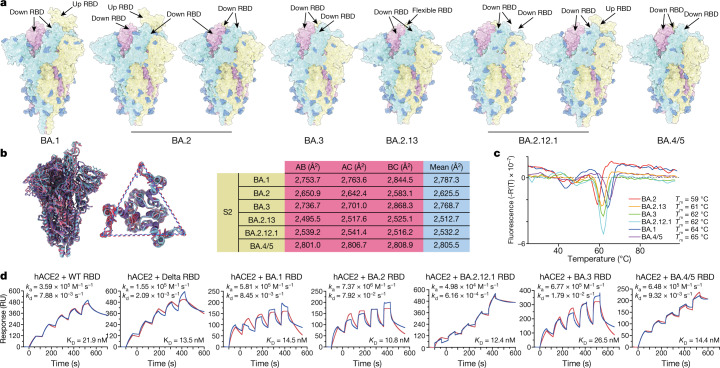
Structural and receptor-binding characteristics of Omicron subvariants. **a**, Surface representation of S-trimers of BA.1, BA.2, BA.3, BA.2.13, BA.2.12.1 and BA.4/BA.5 (BA.4/5) variants. **b**, Structural interpretation and functional verification of the stability of the spike protein of BA.1, BA.2, BA.3, BA.2.13, BA.2.12.1 and BA.4/BA.5 variants. Left, superimposed structures of spike protein and the S2 domains of BA.1 (purple), BA.2 (red) and BA.4/BA.5 (blue). The binding surface areas between S2 subunits of the variants are calculated in the table on the right. **c**, Thermoflour analysis for these Omicron variants. Analyses were performed as biological duplicates. **d**, Binding affinities of RBDs of Omicron variants for hACE2 measured by SPR. Analyses were performed as biological duplicates.

We expressed and purified the prefusion-stabilized trimeric ectodomains of BA.1, BA.2, BA.3, BA.2.12.1, BA.2.13 and BA.4/BA.5 spike (S-trimer). All the S-trimers contain Gly-Ser-Ala-Ser (GSAS) and 6P mutations along with the T4 fibritin trimerization domain for increased stability^14,15^. We determined the cryo-electron microscopy (cryo-EM) structures of these S-trimers at overall resolutions of 3.1–3.5 Å. Together with the previously reported BA.1 structure^16^, this enabled us to compare the structural differences across Omicron sublineages (Fig. 1a and Extended Data Fig. 1b). In contrast to the BA.1 S-trimer, which is stably maintained in an open conformation with one ‘up’ RBD and two ‘down’ RBDs^16^, BA.2 and BA.2.12.1 spike exhibits two conformational states corresponding to a closed form, with all three RBDs in the down configuration and an open form with one RBD in the up position. Of note, one RBD in BA.2.13 was clearly disordered, representing a stochastic movement, which, together with BA.2 and BA.2.12.1, suggests structural heterogeneity in the S-trimers of BA.2 sublineages. Most BA.3 and BA.4 S-trimers adopt closed or semi-closed forms (Fig. 1a). The differences in the RBD up or down conformation could be allosterically modulated by mutations and deletions in the N-terminal domain or near the furin cleavage site, but the detailed mechanism remains unclear. The BA.4/BA.5 spike that we used in our experiments also contains the N658S mutation, which was present in early BA.4/BA.5 sequences but later disappeared owing to the lower transmissibility of this variant, and may correlate with the more closed RBD configurations of the BA.4/BA.5 S-trimer. Of note, S-trimers from the BA.2 sublineage harbour relatively less compact architectures in the region formed by the three copies of S2 (Fig. 1b). By contrast, BA.1, BA.3 and BA.4/BA.5 spike possess relatively tight inter-subunit organization with more buried areas between S2 subunits (Fig. 1b). In line with structural observations, thermal stability assays also verified that S-trimers from BA.2 sublineages were the least stable among these variants, which might confer an enhanced fusion efficiency (Fig. 1c).

Next, we measured the binding affinity between human ACE2 (hACE2) and S-trimers of the Omicron variants by surface plasmon resonance (SPR) (Extended Data Fig. 1c). The BA.4/BA.5 S-trimer showed a decreased binding affinity with hACE2 compared with those of the other Omicron subvariants; however, this measurement could be misleading, owing to the additional N658S mutation. To exclude the potential influence of N658S, we also examined the binding affinities of the RBDs of the Omicron variants for hACE2 (Fig. 1d). The RBDs of Delta (B.1.617.2) and the circulating Omicron subvariants exhibited similar binding affinities for ACE2, except for the BA.3 RBD, which showed a lower affinity, similar to that of the ancestral WT strain. Additionally, the BA.2 subvariants displayed slightly higher binding affinities for hACE2 than the other Omicron variants. To further explore the molecular basis for the altered binding affinities of these variants to hACE2, we performed molecular dynamics simulations based on the cryo-EM structures and examined the effects of substitutions in the RBD on the interaction with hACE2 (Extended Data Fig. 1d). The results reveal that the lack of G496S in BA.2 sublineages meant that the hydrogen bond with hACE2 K353 was regained, increasing their binding capability, in line with experimental observations revealed by deep mutational scanning (DMS) assay^17^. However, a local conformational perturbation surrounding spike residues 444–448 disrupted the hydrophilic interaction between BA.3 spike (S446) with hACE2 Q42, presumably owing to the single mutation G446S rather than double mutations of G446S and G496S (Extended Data Fig. 1d). Notably, the F486V mutation in BA.4/BA.5 spike decreases hACE2 binding activity owing to reduced hydrophobic interaction (Extended Data Fig. 1d). We also noted potential reductions in hydrophilic interactions owing to R493Q reversion. Notably, two reports claimed recently that BA.4/BA.5 RBD and spike (S2P) showed higher binding affinity to hACE2 compared with BA.1 and BA.2 spike, owing to L452R and R493Q reversion^18,19^. Despite this discrepancy, we conclude that BA.2 subvariants and BA.4/BA.5 are able to maintain high binding affinities for hACE2.

## NAb evasion by BA.2.12.1, BA.4 and BA.5

**Fig. 2:**
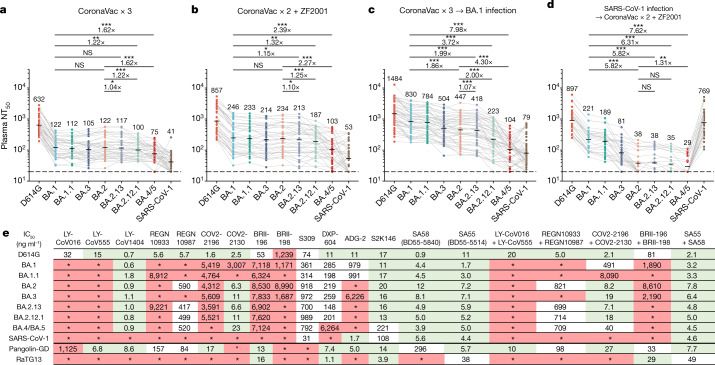
BA.2.12.1, BA.4 and BA.5 exhibit stronger antibody evasion than BA.2. **a**–**d**, Neutralizing titres against SARS-CoV-2 D614G, Omicron subvariants and SARS-CoV-1 pseudoviruses in plasma from vaccinated and convalescent individuals. **a**, Individuals who had received 3 doses of CoronaVac (*n* = 40). **b**, Individuals who had received 2 doses of CoronaVac and 1 dose of ZF2001 (*n* = 38). **c**, Individuals who, after receiving 3 doses of CoronaVac, had been infected with BA.1 and recovered (*n* = 50). **d**, People who had recovered from SARS and received 2 doses of CoronaVac and 1 dose of ZF2001 (*n* = 28). *P*-values were calculated using two-tailed Wilcoxon signed-rank tests of paired samples. The geometric mean titre is shown above each group of points. **e**, Neutralizing activity against SARS-CoV-2 variants and sarbecoviruses by therapeutic NAbs. Green, half-maximal inhibitory concentration (IC_50_) ≤ 30 ng ml^−1^; white, 30 ng ml^−1^ < IC_50_ < 1,000 ng ml^−1^; red, IC_50_ ≥ 1,000 ng ml^−1^; *, IC_50_ ≥ 10,000 ng ml^−1^. All neutralization assays were performed as biological duplicates. *P *<* 0.05, **P *<* 0.01, ***P *<* 0.001; NS, not significant (*P* > 0.05).

**Extended Data Fig. 2:**
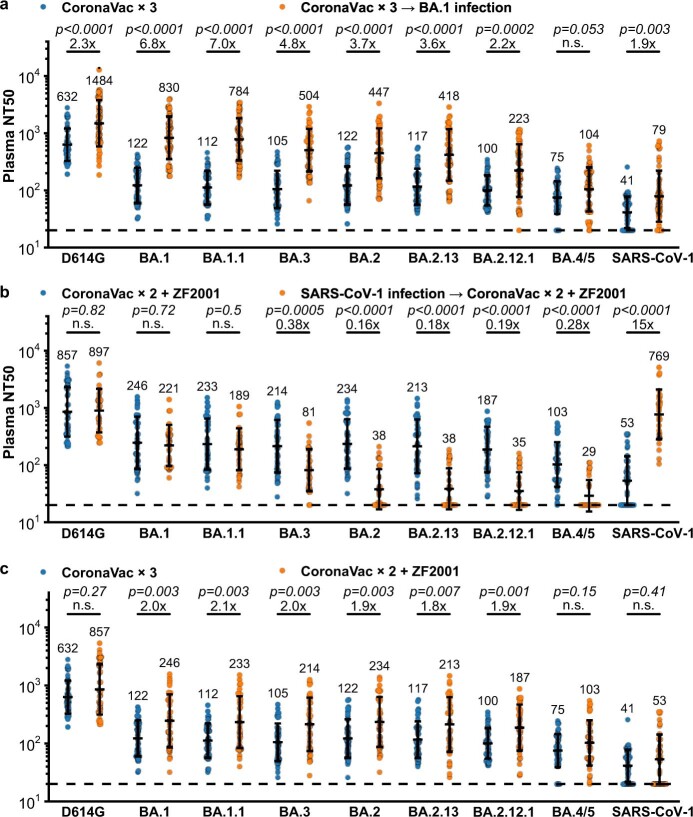
Different immunity backgrounds lead to distinct humoral immunity against Omicron subvariants. NT50 against SARS-CoV-2, SARS-CoV-1 D614G and Omicron subvariants spike-pseudotyped VSV by plasma samples from **a**, individuals who received 3 doses CoronaVac with (n = 50) or without (n = 40) BA.1 breakthrough infection; **b**, individuals who received 2 doses CoronaVac and ZF2001 booster with (n = 28) or without (n = 38) previous SARS-CoV-1 infection; **c**, individuals who received 3 doses CoronaVac (n = 40) or 2 doses CoronaVac with ZF2001 booster (n = 38). P-values were calculated using two-tailed Wilcoxon rank-sum tests and labeled above the bars. n.s., not significant, p > 0.05. All neutralization assays were conducted in biological duplicates. Geometric means are labeled. Error bars refer to geometric standard deviations.

To probe NAb evasion by the recently emerged Omicron sublineages, we performed pseudovirus-neutralization assays using D614G, BA.1, BA.1.1, BA.2, BA.3, BA.2.12.1, BA.2.13 and BA.4/BA.5 against plasma obtained from individuals who had received three doses of SARS-CoV-2 vaccine, vaccinated individuals who had recovered from BA.1 infection, and vaccinated individuals who had recovered from severe acute respiratory syndrome (SARS) (Supplementary Table 1). Plasma samples were collected four weeks after the booster shot or four weeks after discharge from hospital following COVID-19 illness. In plasma from individuals who had received an inactivated virus (CoronaVac) or RBD protein (ZF2001) booster six months after two doses of CoronaVac, BA.1, BA.1.1 and BA.2 showed no significant difference in resistance to neutralization by plasma (Fig. 2a,b), concordant with previous reports^20,21^. However, we found that BA.2 subvariants BA.2.13 and BA.2.12.1 showed increased immune-evasion capability over BA.2—with BA.2.12.1 exhibiting greater evasion than BA.2.13—and BA.4/BA.5 exhibiting even greater evasion (Fig. 2a,b). The decrease in neutralization was clearer in plasma obtained from individuals infected by BA.1 who had received three doses of CoronaVac before infection (Fig. 2c), despite their significantly higher neutralization titres against D614G and BA.1 compared with the triple-dosed vaccinees who had not been infected with BA.1 (Extended Data Fig. 2a). The 50% neutralization titre (NT_50_) in plasma of people who had recovered from BA.1 infection against BA.2.13, BA.2.12.1 and BA.4/BA.5 was reduced by 2.0-, 3.7- and 8-fold, respectively, compared with that for BA.1. Plasma from vaccinated individuals who had recovered from SARS infection showed a marked decrease in neutralization of BA.2 subvariants, BA.3 and BA.4/BA.5 compared with the other vaccinees (Fig. 2d and Extended Data Fig. 2b). This suggests that mutations in BA.2 sublineages, BA.3 and BA.4/BA.5 may enable escape from broad sarbecovirus-neutralizing antibodies, which are substantially enriched in vaccinated people previously infected with SARS^22^. Together, these observations indicate that the BA.2.12.1 and BA.4/BA.5 display more potent and distinct humoral immune evasion than BA.1.

Next, we examined the neutralizing activities of therapeutic antibodies against new Omicron subvariants (Fig. 2e). All seven tested Omicron subvariants displayed substantial evasion against neutralization by class 1 and class 2 RBD antibodies: the variants evaded neutralization by REGN-10933^23^ (casirivimab), LY-CoV016^24^ (etesevimab), LY-CoV555^25^ (bamlanivimab), COV2-2196^5^ (tixagevimab) and BRII-196^26^ (amubarvimab), whereas only BA.4/BA.5 evaded DXP-604^15,27^, which showed reduced but still competitive efficacy against BA.1 and BA.2 subvariants. Two major differences in antigenicity were observed between BA.1 and BA.2 subvariants. First, NAbs targeting the linear epitope 440–449^3^, such as REGN-10987^23^ (imdevimab), COV2-2130^5^ (cilgavimab, a component of Evusheld) and LY-CoV1404 (bebtelovimab^4^) could neutralize BA.2 subvariants and BA.4/BA.5. Second, BA.2 sublineages greatly reduced the efficacy of BA.1-effective broad sarbecovirus-neutralizing antibodies, including ADG-2^28^ (adintrevimab) and S309^29^ (sotrovimab), but not the ACE2-mimicking antibody S2K146^30^, which potently neutralized all BA.1 and BA.2 sublineages but showed reduced activity against BA.4/BA.5, similar to DXP-604. BA.2 sublineages^9^, BA.3 and BA.4/BA.5 escaped neutralization by a BRII-196 and BRII-198 cocktail (amubarvimab plus romlusevimab). Notably, LY-CoV1404^4^ demonstrated high potency against all tested Omicron subvariants. In addition, our recently developed non-competing antibody cocktail comprising two NAbs isolated from vaccinated individuals who had recovered from SARS, namely BD55-5840 (also known as SA58; class 3) and BD55-5514 (also known as SA55; class 1/4), displayed high potency against the Omicron subvariants and the sarbecoviruses SARS-CoV-1, Pangolin-GD and RaTG13.

**Fig. 3:**
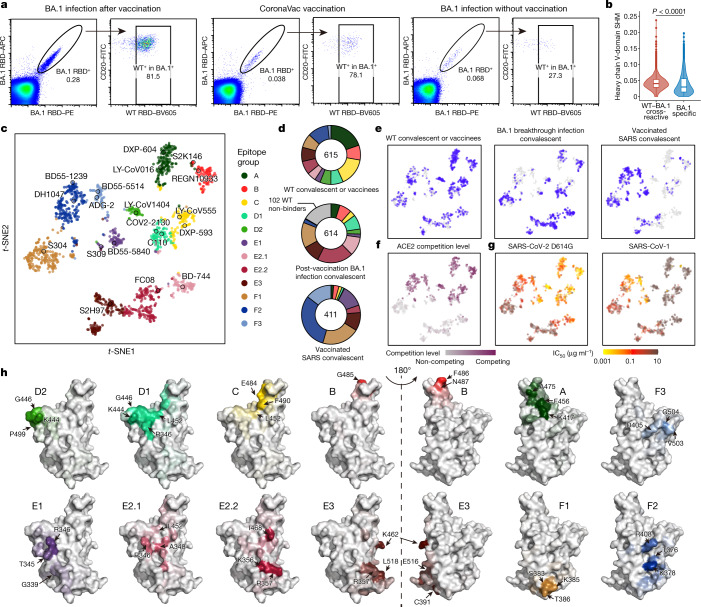
Isolation, characterization, and comprehensive epitope mapping of SARS-CoV-2 RBD antibodies. **a**, FACS analysis of pooled memory B cells (IgM^−^CD27^+^) from plasma of individuals who have recovered from BA.1 breakthrough infection after vaccination, vaccinated individuals and unvaccinated individuals who have recovered from BA.1 breakthrough infection. The percentage of cells recognizing WT or BA.1 RBD are shown. **b**, The heavy chain V domain somatic hypermutation (SHM) rate of BA.1-specific (*n* = 968) and BA.1–WT cross-reactive (*n* = 4,782) BCRs obtained from 10X scVDJ-seq from individuals who have recovered from BA.1 breakthrough infection after vaccination. Two-tailed Wilcoxon rank-sum test. Boxes show 25th percentile, median and 75th percentile, and violin plots show kernel density estimation curves of the distribution. **c**, *t*-SNE and unsupervised clustering of antibodies that bind WT SARS-CoV-2 RBD. Twelve epitope groups were identified on the basis of DMS of 1,538 antibodies. **d**,**e**, Epitope distribution and projection of antibodies from plasma of individuals who had recovered from infection with the WT virus, individuals who have recovered from BA.1 breakthrough infection after vaccination, and vaccinated individuals who had recovered from SARS. **f**, ACE2 competition level determined by competition ELISA (*n* = 1,286) were projected onto the *t*-SNE. **g**, Neutralizing activity against SARS-CoV-2 D614G (*n* = 1,509) and SARS-CoV-1 (HKU-39849; *n* = 1,457). **h**, Average mutational escape score projection of each epitope group on SARS-CoV-2 RBD (Protein Data Bank (PDB): 6M0J). All neutralization assays were performed as biological duplicates.

**Extended Data Fig. 3:**
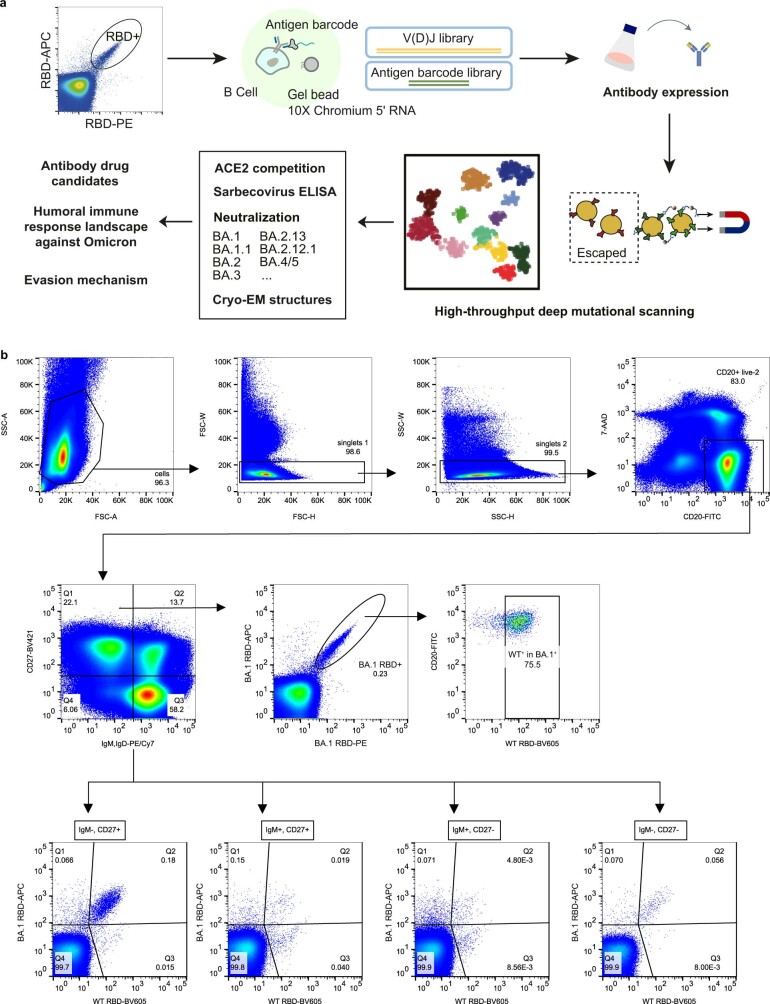
Workflow for the isolation and characterization of SARS-CoV-2 RBD antibodies. **a**, Overall schematic of antibody identification by single cell VDJ sequencing with feature barcodes and epitope analysis by high-throughput deep mutational scanning. **b**, FACS strategy to enrich BA.1/WT cross-reactive memory B cells or BA.1-specific memory B cells.

To delineate the underlying antibody-evasion mechanism of BA.2.13, BA.2.12.1 and BA.4/BA.5, in particular the escape from the humoral immunity induced by recovery from BA.1 infection or recovery from SARS in vaccinated individuals, we began by isolating RBD-targeting NAbs from such individuals^27,31^ (Extended Data Fig. 3a). We isolated antigen-specific memory B cells by fluorescence-activated cell sorting (FACS) of pooled peripheral blood mononuclear cells (PBMCs) using double WT RBD^+^ selection for triple-vaccinated people, WT RBD^+^ and SARS-CoV-1 RBD^+^ selection for vaccinated individuals who had recovered from SARS and double BA.1 RBD^+^ selection for individuals who had recovered from BA.1 infection (Extended Data Fig. 3b). We then performed single-cell V(D)J sequencing (scVDJ-seq) with BA.1 RBD and WT RBD feature barcodes on CD27^+^IgM^−^ antigen-specific memory B cells (Extended Data Fig. 3b). We also extracted the productive heavy-light chain paired V(D)J sequences and expressed the antibodies in vitro as human IgG1. We found that the majority of Omicron-reactive memory B cells from triple-CornonaVac-vaccinated individuals who had recovered from BA.1 infection could also bind to WT RBD (Fig. 3a). By contrast, only around a quarter of Omicron-reactive memory B cells isolated from unvaccinated individuals who had recovered from BA.1 infection could bind to WT RBD (Fig. 3a). The cross-reactive antigen-binding property was observed only in IgM^−^CD27^+^ memory B cells, but not in IgM^+^CD27^−^ naive B cells (Extended Data Fig. 2b). V(D)J sequence analysis revealed significantly higher heavy chain V-domain somatic hypermutation rates of BA.1 and WT cross-reactive B cell receptors (BCRs) than that of BA.1-specific BCRs (Fig. 3b), which indicates that the cross-reactive memory B cells were further affinity-matured compared with BA.1-specific memory B cells. Together, these data suggest that post-vaccination infection with Omicron BA.1 recalls mainly WT-induced memory B cells.

To further specify the epitope distribution of NAbs elicited by post-vaccination BA.1 infection, we applied high-throughput yeast display-based DMS assays^2,3^ and determined the mutation profiles for escape for 1,640 RBD-binding antibodies. Among these antibodies, 602 were from individuals who had recovered from WT SARS-CoV-2 or triple vaccinees, 614 were from individuals who had recovered from post-vaccination BA.1 infection, and 410 SARS–WT SARS-CoV-2 cross-reactive antibodies were from vaccinated individuals who had recovered from SARS (Supplementary Table 2). We also included 14 antibodies with published DMS profiles in this analysis^2,32,33^. Of note, among the 614 antibodies from individuals who had recovered from post-vaccination BA.1 infection, 102 were BA.1-specific and did not bind to the WT RBD. The escaping mutation profiles of these 102 BA.1-specific NAbs were determined by DMS based on the BA.1 RBD. The remaining 1,538 WT RBD-reactive antibodies were sorted by unsupervised clustering into 12 epitope groups according to their WT-based mutational escape profiles using *t*-distributed stochastic neighbour embedding (*t*-SNE) (Fig. 3c), adding 6 additional epitope groups to our previous classification^3^.

**Extended Data Fig. 4:**
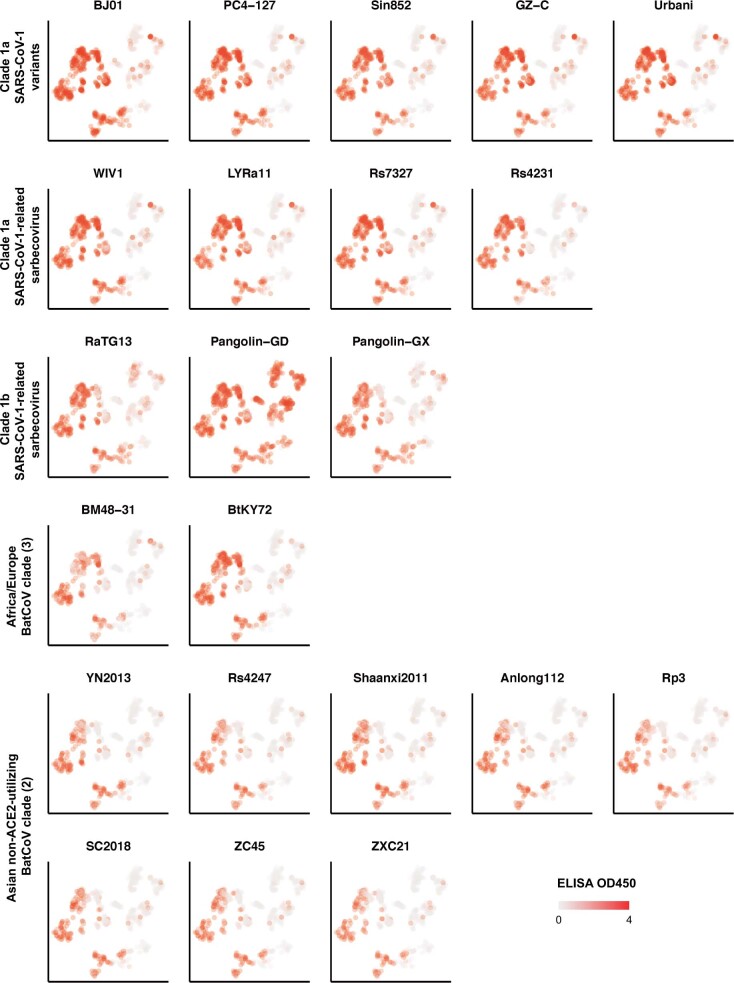
ELISA reactivity against 22 sarbecovirus RBD. Shades of red indicate ELISA OD450 for each antibody against various sarbecoviruses from different clades.

Groups A–C recapitulate our previous taxonomy^3^ in which the members target mainly the ACE2-binding motif^34–38^ (Fig. 3h). Group D antibodies, such as REGN-10987, LY-CoV1404 and COV2-2130, bind to the linear epitope 440–449 on the RBD and are divided into D1 and D2 subgroups. Group D1 is more affected by mutations of R346 and L452, whereas D2 antibodies are not and interact more with P499 (Fig. 3h). Additionally, groups E and F are divided into E1–E3 and F1–F3, covering the front and back of the RBD and roughly corresponding to class 3 and class 4 antibodies, respectively^37^ (Fig. 3h). Group E1 occupies the S309 binding site, whose epitope involves G339, T345 and R346. Group E2 antibodies bind to the front chest of RBD^35^. Group E2.1 binding is most affected by mutations of R346 and A348, whereas E2.2 is most affected by K356 and R357. Groups E3 (S2H97 site) and F1 (S304 site) bind to highly conserved regions on the bottom of the RBD, interacting mainly with K462/E516/L518 and S383/T385/K386, respectively. Antibodies in groups E1–E3 and F1 do not compete with ACE2 (Fig. 3f), whereas F2 and F3 antibodies compete with ACE2 and their binding is affected by T376, K378, D405, R408 and G504, corresponding to class 1/4^39^. We tested the pseuodovirus-neutralizing efficacy of antibodies in each group against SARS-CoV-1, SARS-CoV-2 D614G, Pangolin-GD and RaTG13, as well as their ability to bind to 22 sarbecovirus RBDs using ELISA (Supplementary Tables 2 and  3). Antibodies within the same cluster shared a common sarbecovirus neutralization potency and binding spectrum (Fig. 3g and Extended Data Fig. 4). In total, we identified five clusters of antibodies exhibiting broad sarbecovirus-binding ability, namely groups E1, E3, F1, F2 and F3 (Extended Data Fig. 4), and antibodies in groups E1, F2 and F3 showed potent neutralizing activity against SARS-CoV-1 (Fig. 3g).

Of note, we found that plasma from individuals who had recovered from post-vaccination BA.1 infection displayed enrichment of group E2.1, E2.2 and F1 antibodies (Fig. 3d,e), which do not compete for binding with ACE2 (Fig. 3f). BA.1 does not harbour mutations on the epitopes of these NAb groups, which may explain why post-vaccination BA.1 infection is more likely to stimulate those NAbs. Although they are not enriched, the ACE2-competing group B and D1 antibodies remain highly abundant after infection. Since group E2, D1 and B antibodies are sensitive to mutation at residues 452 and 486 (Fig. 3h), it is highly probable that BA.2.12.1, BA.2.13 and BA.4/BA.5 can specifically target those antibodies, rationalizing the large decrease in NT_50_ of plasma from individuals who have recovered from BA.1 infection against those variants (Fig. 2c).

**Extended Data Fig. 5:**
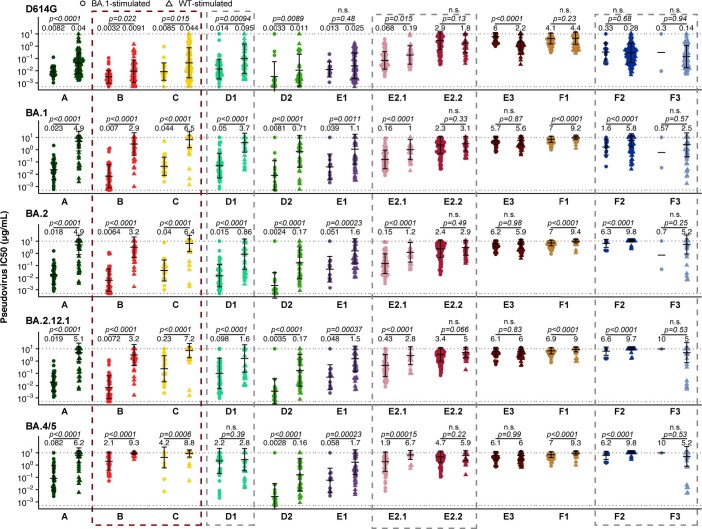
Neutralizing activities of antibodies elicited by SARS-CoV-2 BA.1 or wildtype. Neutralizing activity against SARS-CoV-2 D614G and Omicron subvariants pseudovirus by antibodies of each epitope group from BA.1 convalescents (BA.1-stimulated. A, n = 30; B, n = 41; C, n = 20; D1, n = 49; D2, n = 17; E1, n = 11; E2.1, n = 64; E2.2, n = 122; E3, n = 57; F1, n = 80; F2, n = 13; F3, n = 2), and from wildtype convalescents or vaccinees (WT-stimulated. A, n = 98; B, n = 55; C, n = 88; D1, n = 46; D2, n = 36; E1, n = 59; E2.1, n = 26; E2.2, n = 39; E3, n = 68; F1, n = 97; F2, n = 158; F3, n = 67). Geometric mean titers (GMT) are annotated above each group of points, and error bars indicate geometric standard deviation. P-values were calculated using two-tailed Wilcoxon rank-sum tests and labeled above the bars. n.s., not significant, p > 0.05. NAbs in the boxed epitope groups showed substantial neutralization potency changes against BA.2.12.1 or BA.4/5 compared to BA.1. All neutralization assays were conducted in biological duplicates.

To examine our hypothesis, we measured pseudovirus neutralization of these NAbs against BA.2.12.1, BA.2.13 and BA.4/BA.5, as well as the major Omicron variants BA.1, BA.1.1, BA.2 and BA.3 (Extended Data Fig. 5). NAbs from different epitope groups displayed distinct neutralizing activities against Omicron subvariants. BA.1-stimulated antibodies (following recovery from BA.1 infection) and WT-stimulated (following recovery from WT infection or vaccination, with or without previous SARS-CoV-1 infection) showed significantly higher potency and breadth in most epitope groups, confirming the increased affinity maturation of these antibodies (Extended Data Fig. 5).

**Extended Data Fig. 6:**
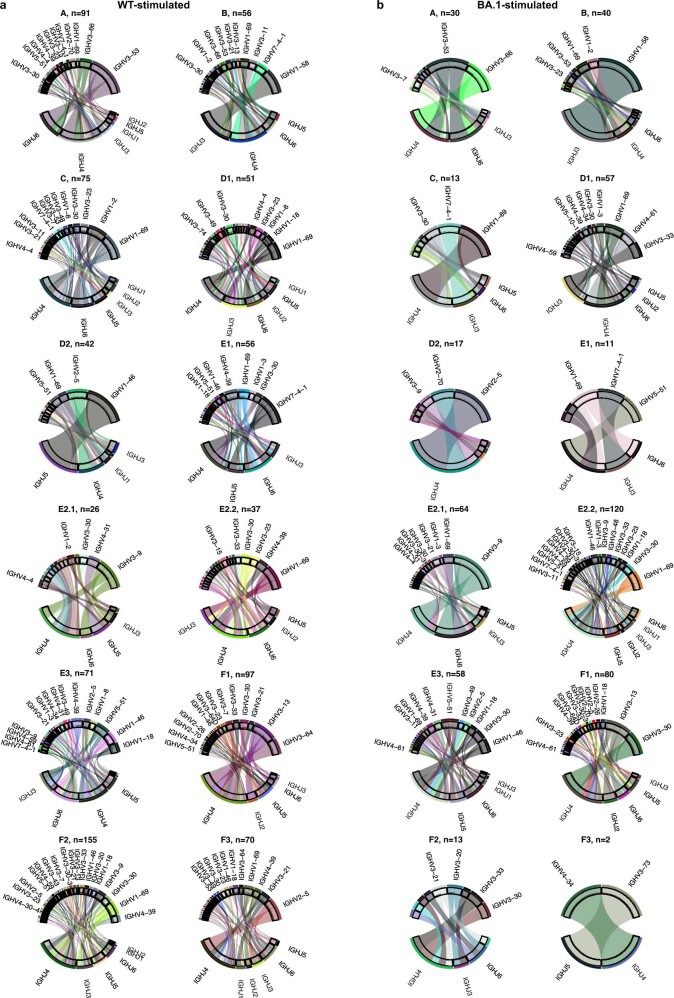
Heavy chain V-J genes of BA.1-stimulated and WT-stimulated antibodies in each epitope group. Heavy chain V-J genes combination of **a**, WT-stimulated antibodies. **b**, BA.1-stimulated antibodies or each epitope group. The number of NAbs is annotated above the chord plot. IGHV genes are annotated only if the corresponding number of antibodies is greater than one.

**Extended Data Fig. 7:**
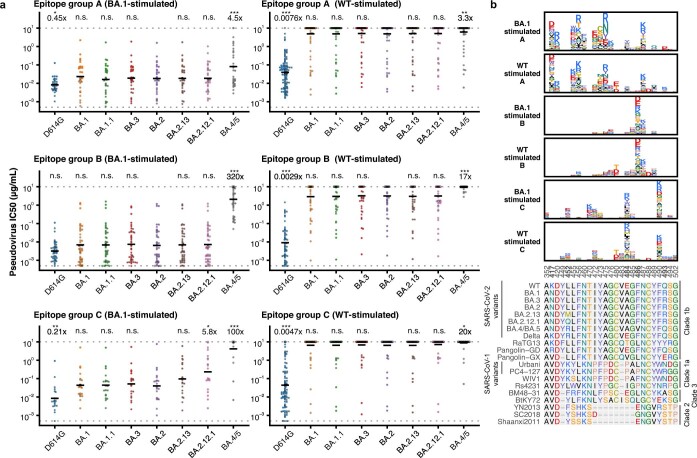
Comparison of BA.1-stimulated and WT-stimulated antibodies in group A, B and C. **a**, Neutralizing activity against SARS-CoV-2 D614G and Omicron subvariants by BA.1-stimulated (A, n = 30; B, n = 41; C, n = 20) and WT-stimulated (A, n = 98; B, n = 55; C, n = 88) antibodies in Group A, B and C. Geometric mean of IC50 fold changes compared to IC50 against BA.2 are annotated above the bars. P-values were calculated using a two-tailed Wilcoxon signed-rank test of paired samples, in comparison to IC50 against BA.2. *, p < 0.05; **, p < 0.01; ***, p < 0.001; n.s., not significant, p > 0.05. All neutralization assays were conducted in biological duplicates. **b**, Averaged escape maps at escape hotspots of BA.1-stimulated and WT-stimulated antibodies in group A, B and C, and corresponding MSA of various sarbecovirus RBDs. Height of each amino acid in the escape maps represents its mutation escape score. Mutated sites in Omicron variants are marked in bold.

Omicron subvariants evaded most WT-stimulated group A, B and C NAbs, although a subset of these antibodies showed broad effectiveness against Omicron (Extended Data Fig. 5). These broad NAbs were largely enriched by BA.1 stimulation, are generally encoded by similar heavy chain V genes compared with WT-stimulated antibodies and display higher convergence (Extended Data Fig. 6a,b). These broad ACE2-competing NAbs in groups A, B and C have been shown to be enriched in individuals who received a booster dose of mRNA vaccine^39^, which probably accounts for the high neutralizing activity against Omicron variants in plasma of individuals who had received three doses of mRNA vaccine. Nevertheless, BA.1-stimulated group B and C NAbs were significantly evaded by BA.4 owing to F486V and L452R RBD mutations, concordant with results from DMS (Extended Data Fig. 7a,b), which explains the strong humoral immune-evasion ability of BA.4/BA.5.

**Fig. 4:**
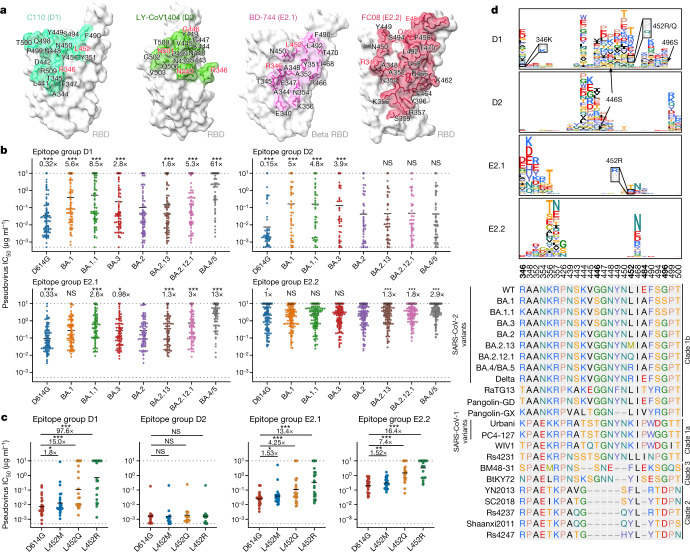
Spike L452 mutants can evade cross-reactive NAbs elicited by BA.1 infection. **a**, Epitope of representative antibodies in group D1 (C110; PDB: 7K8V), D2 (LY-CoV1404; PDB: 7MMO), E2.1 (BD-744; PDB: 7EY0) and E2.2 (FC08; PDB: 7DX4). Residues highlighted in red indicate sites that are mutated in Omicron variants. **b**, Neutralizing activity of NAbs in group D1 (*n* = 95), D2 (*n* = 53), E2.1 (*n* = 90) and E2.2 (*n* = 161) against spike-pseudotyped SARS-CoV-2 variants. The geometric mean of the fold change in IC_50_ relative to BA.2 is shown above each plot. Two-tailed Wilcoxon signed-rank test of paired samples, in comparison to IC_50_ values versus BA.2. **c**, Neutralizing activity of representative potent NAbs in group D1 (*n* = 24), D2 (*n* = 12), E2.1 (*n* = 23) and E2.2 (*n* = 23) against SARS-CoV-2 spike L452 mutants. Geometric mean of the fold change in IC_50_ relative to D614G is shown above each plot. Two-tailed Wilcoxon signed-rank test of paired samples. **d**, Average escape maps at escape hotspots of antibodies in epitope groups D1, D2, E2.1 and E2.2, and the corresponding multiple sequence alignment of various sarbecovirus RBDs. The height of each amino acid in the escape map represents its mutation escape score. Sites that are mutated in Omicron subvariants are marked in bold. All neutralization assays were performed as biological duplicates.

Group D antibodies were most affected by the G446S mutation in BA.1, BA.1.1 and BA.3 (Fig. 4d); these NAbs therefore show higher potency against BA.2 (Fig. 4a,b). However, group D1 antibodies showed reduced efficacy against L452 substitutions, with L452M (BA.2.13) causing mild escape, L452Q causing moderate escape (BA.2.12.1) and L452R (BA.4/BA.5) causing severe escape (Fig. 4c,d). By contrast, group D2 antibodies, especially those stimulated by BA.1 infection, showed exceptional broad and potent neutralizing activity against all Omicron subvariants, for example, LY-CoV1404 (Fig. 4b and Extended Data Fig. 5). Notably, although group D2 NAbs displayed broad activities, their epitopes are not conserved among sarbecoviruses (Fig. 4d), similar to those of group D1, E2.1 and E2.2 NAbs. This suggests that their breadth may be a result of their rarity in individuals who have recovered from infection with WT or BA.1 SARS-CoV-2 (Fig. 3f), and these NAbs may be the next target for SARS-CoV-2 to escape by evolving specific mutations on their epitopes.

Group E2 antibodies bind to the chest of the RBD^35^ (Fig. 4a), and their epitopes are focused around R346, A348, A352, K356, R357 and I468 (Fig. 4d). Despite similar epitopes, group E2.1 NAbs, especially those stimulated by BA.1, show significantly higher neutralizing potency than group E2.2 NAbs (Fig. 4b). E2 group NAbs showed broad specificity against SARS-COV-2 variants but not against BA.2.12.1 and BA.4/BA.5. L452 substitutions can result in large-scale escape of E2.1 and E2.2 antibodies (Fig. 4c). Similar to the D1 epitope group, L452R and L452Q resulted in considerably increased NAb evasion over L452M (Fig. 4c). Of note, DMS does not indicate the sensitivity of the E2.2 epitope group to L452 substitution (Fig. 4d). Together, our results suggest that Omicron may have evolved mutations at L452 to specifically evade group D1 and E2 NAbs, consequently maximizing humoral immune evasion after infection with Omicron BA.1. Of note, group D1 and E2.1 antibodies also showed decreased efficacy against BA.1.1 compared with BA.1 (Fig. 4b) as a result of the R346K mutation, since both groups of NAbs are sensitive to the R346 substitution (Fig. 4a,d), suggesting a reason for the high prevalence of BA.1.1 in the population after the BA.1 wave in the United States.

## Omicron escapes broad sarbecovirus NAbs

**Fig. 5:**
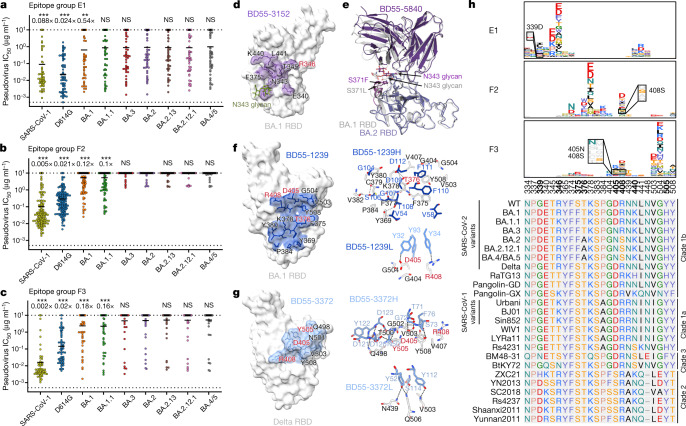
BA.2 subvariants can escape most broad-specificity sarbecovirus-neutralizing antibodies. **a**–**c**, Neutralizing activity against SARS-CoV-1 and SARS-CoV-2 subvariants by NAbs in group E1 (**a**; *n* = 70), F2 (**b**; *n* = 171) and F3 (**c**; *n* = 69). The geometric mean of the fold change in IC_50_ relative to BA.2 is shown above each plot. *P*-values were calculated using a two-tailed Wilcoxon signed-rank test of paired samples, compared with the IC_50_ for BA.2. **d**, The epitope of Group E1 antibody BD55-3152 on the BA.1 RBD. **e**, Overlay of BD55-5840 in the complex with BA.1 or BA.2 RBD. **f**,**g**, The epitope and interactions on the binding interface of BD55-1239 (group F2) (**f**) and BD55-3372 (group F3) (**g**). Antibody residues are shown in blue, and RBD residues are in black or red. Residues highlighted in red indicate sites that are mutated in Omicron variants. **h**, Average escape maps of antibodies in epitope groups E1, F2 and F3, and the corresponding multiple sequence alignment of various sarbecovirus RBDs. The height of each amino acid in the escape map represents its mutation escape score. Sites that are mutated in Omicron subvariants are marked in bold. All neutralization assays were performed as biological duplicates.

**Extended Data Fig. 8:**
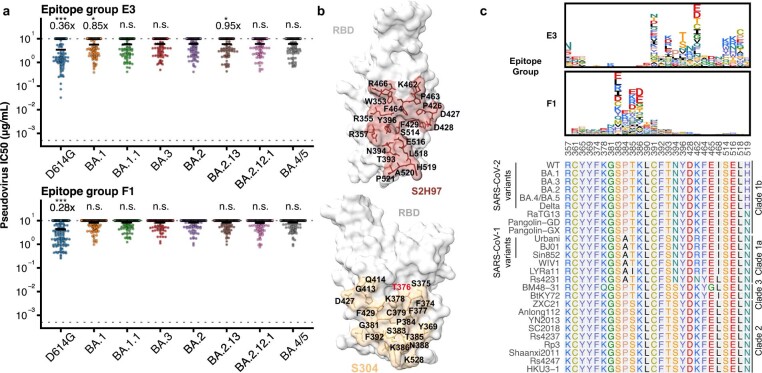
Antibodies of group E3 and F1 exhibit weak but broad-spectrum neutralization. **a**, Neutralizing activity against SARS-CoV-2 D614G and Omicron subvariants by antibodies in group E3 (n = 125) and F1 (n = 177). Geometric mean of IC50 fold changes compared to BA.2 are annotated above the bars. P-values were calculated using a two-tailed Wilcoxon signed-rank test of paired samples, in comparison to IC50 against BA.2. *, p < 0.05; **, p < 0.01; ***, p < 0.001; n.s., not significant, p > 0.05. All neutralization assays were conducted in biological duplicates. **b**, Epitope of representative antibodies in group E3 (S2H97, PDB: 7M7W) and F1 (S304, PDB: 7JW0). Residues highlighted in red indicate mutated sites in Omicron variants. **c**, Averaged escape maps at escape hotspots of antibodies in group E3 and F1, and corresponding MSA of various sarbecovirus RBDs. Height of each amino acid in the escape maps represents its mutation escape score. Mutated sites in Omicron variants are marked in bold.

In total, five clusters of antibodies were found to exhibit broad sarbecovirus-neutralizing ability with diverse specificity, namely groups E1, E3, F1, F2 and F3 (Extended Data Fig. 4). Whereas Group E3 and F1 antibodies demonstrated weak neutralizing activity against all variants owing to their highly conserved binding sites (Extended Data Fig. 8a–c), we found that E1, F2 and F3 NAbs—which are effective against BA.1, were rare in individuals after infection with WT SARS-CoV-2 or Omicron but enriched in vaccinated individuals who had recovered from SARS infection—displayed a systematic reduction in neutralization activity against BA.2 subvariants, BA.3 and BA.4/BA.5 (Figs. 2e and 5a–c). This observation explains the low NT_50_ for Omicron subvariants other than BA.1 in plasma from individuals who had recovered from SARS infection (Fig. 2d). The mechanisms behind the loss of neutralization by these broad-specificity sarbecovirus antibodies require investigation, as they may prove to be crucial for developing broad-spectrum sarbecovirus vaccines and antibody therapies.

**Extended Data Fig. 9:**
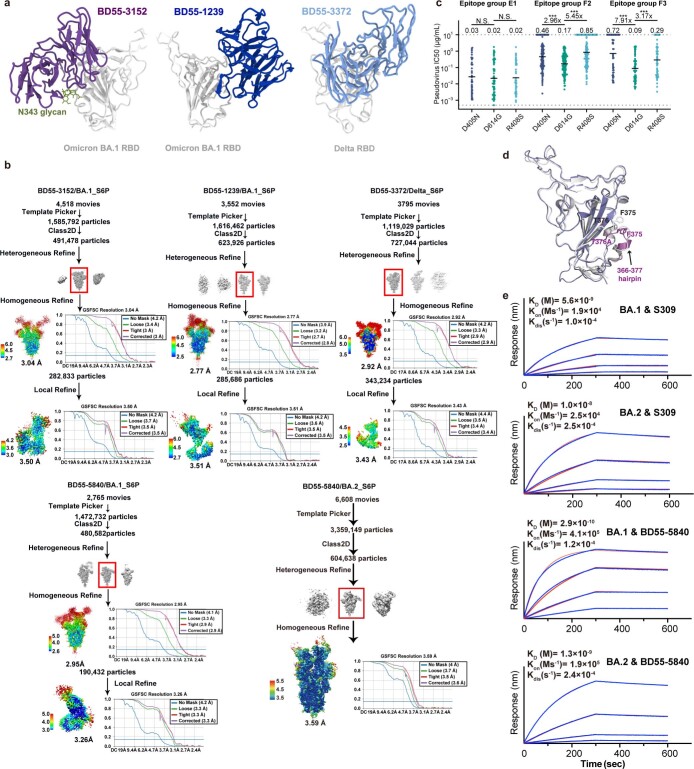
RBD-binding structures and affinity of broad Sarbecovirus antibodies. **a**, Cartoon models of Cryo-EM structures of BD55-3152 in complex of BA.1 RBD, BD55-1239 in complex of BA.1 RBD, and BD55-3372 in complex of Delta RBD. **b**, Workflow to generate refined structural model of BD55-3152 and BD55-1239 in complex of BA.1 RBD, BD55-3372 in complex of Delta RBD, and BD55-5840 in complex of BA.2 RBD. **c**, Neutralizing activity of representative NAbs in group E1 (n = 68), F2 (n = 139) and F3 (n = 61) against SARS-CoV-2 D614G, in addition to D614G+D405N and D614G+R408S. Geometric mean of IC50 fold changes compared to IC50 against D614G are annotated above the bars. P-values were calculated using a two-tailed Wilcoxon signed-rank test of paired samples. *, p < 0.05; **, p < 0.01; ***, p < 0.001; n.s., not significant, p > 0.05. All neutralization assays were conducted in biological duplicates. **d**, Conformational comparison between BA.1 and BA.2 RBD regarding the 366-377 hairpin. **e**, Biolayer interferometry analysis of Group E1 antibodies S309 and BD55-5840 binding to Omicron BA.1 and BA.2 Spike trimer. Biolayer interferometry analyses were conducted in biological duplicates.

To study how BA.2 subvariants, BA.3 and BA.4/BA.5 could systematically reduce the neutralization efficacy of group E1 antibodies, we solved the cryo-EM structures of two group E1 BA.1-neutralizing antibodies, BD55-3152 and BD55-5840, in complex with BA.1 spike proteins using cryo-EM (Fig. 5d and Extended Data Fig. 9a,b). Similar to S309, the epitope of group E1 antibodies includes an *N*-linked glycan on N343 (Fig. 5d). Group E1 antibodies are also generally sensitive to mutation of G339, E340, T345 and especially R346, as indicated by their escaping mutation profiles (Fig. 5h). Notably, the newly acquired mutations of BA.2 do not overlap with the shared epitope of E1 antibodies, suggesting that the systematic reduction in neutralization is not caused by amino acid substitution and is potentially owing to structural alteration. To explore this hypothesis, we further determined the cryo-EM structure of the prefusion-stabilized BA.2 spike in complex with the BD55-5840 Fab (Fig. 5e). A structural comparison with the BA.1 RBD binding to BD55-5840 described above suggests that the 366–377 hairpin loop displays significant conformational differences due to S371F and T376A mutations (Fig. 5e and Extended Data Fig. 9d). The overall positions of residues 375 and 376 are displaced by more than 3 Å, which probably further decreases the binding of group F2 and F3 NAbs in addition to the T376A side-chain substitution. As a result, the bulky phenylalanine resulting from the S371F mutation interferes with the positioning of the glycan moiety attached to N343, which in turn shifts the heavy chain of BD55-5840 upward. This may explain the decreased binding between BD55-5840 and S309, rationalizing their reduced neutralizing activity (Fig. 5a and Extended Data Fig. 9e). The N343 glycan is critically recognized by almost all group E1 NAbs, including S309. Thus, this group of broad and potent NAbs is probably affected by the S371F mutation in a systematic manner through displacement of the N343 glycan.

The epitopes of group F2 and F3 antibodies cover a continuous surface on the back of the RBD and can only bind to RBDs in the up configuration (Fig. 2b). To probe how BA.2 escapes group F2 and F3 antibodies, we solved the cryo-EM structure of two representative BA.1-neutralizing antibodies—BD55-1239 from group F2 and BD55-3372 from group F3—in complex with BA.1 and Delta spike protein, respectively (Fig. 5f,g and Extended Data Fig. 9a). RBD mutations on T376, K378 and R408 can lead to escape from neutralization by group F2 antibodies (Fig. 5h). Indeed, these residues are centred on the core of the BD55-1239 epitope and are fairly conserved across sarbecoviruses (Fig. 5h). Notably, D405N and R408S, which are present in Omicron BA.2 sublineages, may alter the antigenic surface, disrupting the binding of F2 antibodies (Fig. 5f) and completely abolishing the neutralizing capacity of F2 antibodies (Fig. 5b). Similarly, the D405N and R408S mutations harboured by BA.2 subvariants could interrupt the heavy chain binding of F3 antibodies, causing large-scale escapes of BA.1-neutralizing group F3 NAbs (Fig. 5c). These observations were further validated by neutralizing activity against spike-pseudotyped vesicular stomatitis virus (VSV) harbouring D614G/D405N and D614G/R408S. As expected, group E1 antibodies were not affected, whereas group F2 and F3 antibodies displayed significantly decreased activity, following D405N or R408S single substitutions (Extended Data Fig. 9c). Nevertheless, several group F3 antibodies, such as BD55-5514, are not sensitive to the D405N and R408S mutations of BA.2, making them good therapeutic drug candidates (Fig. 2e). In sum, S371F, D405N and R408S mutations harboured by BA.2 and emerging Omicron variants may induce large-scale escape of NAbs with broad sarbecovirus specificity, which are critical for the development of broad-specificity sarbecovirus antibody therapies and vaccines.

## BA.1-specific NAbs exhibit narrow breadths

**Fig. 6:**
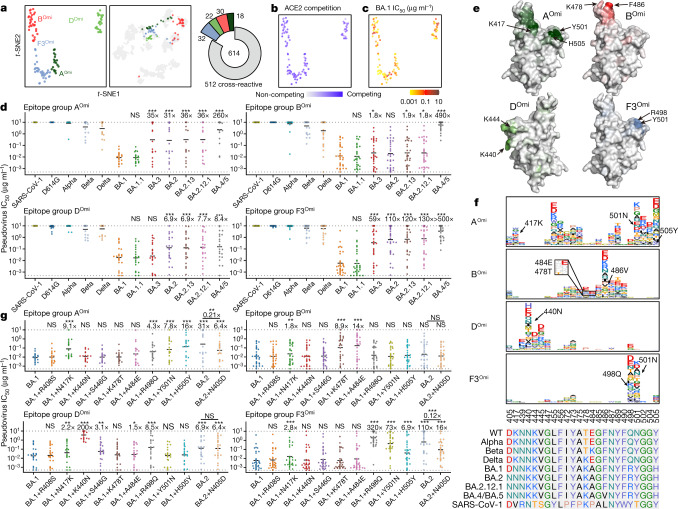
BA.1-specific antibodies elicited by BA.1 infection exhibit narrow specificity. **a**, Four epitope groups were identified among 102 BA.1-specific NAbs via *k*-means clustering and *t*-SNE of BA.1 RBD-based DMS profiles. **b**,**c**, Distribution of ACE2 competition level (**b**) and neutralizing activities (**c**) against BA.1. **d**, Neutralizing activities of BA.1-specific antibodies against pseudovirus with SARS-CoV-1 and SARS-CoV-2 spike variants (A^Omi^, *n* = 18; B^Omi^, *n* = 30; D^Omi^, *n* = 22; F3^Omi^, *n* = 32). The geometric mean of the fold change in IC_50_ relative to BA.1 is shown above each plot. **e**, Average mutational escape score projection of each BA.1-specific epitope group on SARS-CoV-2 RBD (PDB: 7WPB). **f**, Averaged escape maps at escape hotspots of the 102 NAbs in the four epitope groups, and corresponding multiple sequence alignment of various sarbecovirus RBDs. The height of each amino acid in the escape map represents its mutation escape score. Sites that are mutated in Omicron variants are marked in bold. WT-related escaping mutations are highlighted. **g**, Neutralizing activities of BA.1-specific NAbs against BA.1- or BA.2-based pseudoviruses carrying single substitutions (A^Omi^, *n* = 18; B^Omi^, *n* = 30; D^Omi^, *n* = 22; F3^Omi^, *n* = 32). The geometric mean of the fold change in IC_50_ relative to BA.1 is shown above each plot. Wilcoxon signed-rank test of paired samples, compared with IC_50_ for BA.1. All neutralization assays were performed as biological duplicates.

**Extended Data Fig. 10:**
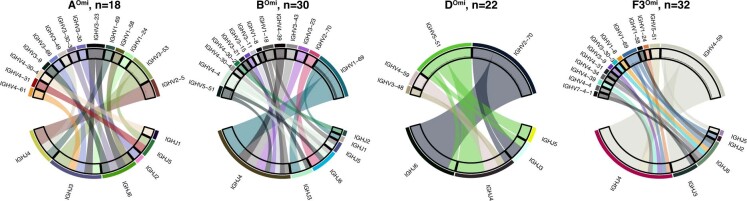
HV-HJ gene combination of BA.1-specific antibodies. Heavy chain V-J gene combination of BA.1-specific neutralizing antibodies in BA.1-specific epitope groups A^Omi^, B^Omi^, D^Omi^ and F3^Omi^.

In addition to the WT–BA.1 cross-reactive NAbs, we also investigated the epitope distribution of BA.1-specific NAbs that do not react with WT RBD. We built a yeast display variants library based on the BA.1 RBD, and determined the escape mutation maps of 102 BA.1-specific antibodies. By integrating the analysis of the entire dataset of 1,640 SARS-CoV-2 RBD antibodies, we derived the embedded features of the BA.1-specific NAbs and performed clustering and *t*-SNE analysis (Fig. 6a). The 102 NAbs were clustered into four BA.1-specific epitope groups, which we designated A^Omi^, B^Omi^, D^Omi^ and F3^Omi^, since these groups are closely related to the corresponding WT epitope groups (Fig. 6a,e). These antibodies all compete for binding with ACE2 and potently neutralize BA.1 but do not neutralize SARS-CoV-2 D614G or SARS-CoV-1 (Fig. 6b–d) because of the differences in the spike protein: N417K/Y501N/H505Y for A^Omi^, A484E/K478T for B^Omi^, K440N for D^Omi^ and R498Q/Y501N for F3^Omi^, as indicated by average escape maps of each group (Fig. 6e,f). Some of the previously circulating variants also harbour these same mutations—such as N501Y in Alpha (B.1.1.7), K417N/E484K/N501Y in Beta (B.1.351) and T478K in Delta—and only a small subset of the antibodies exhibit neutralizing activity against these variants (Fig. 6e). Moreover, nearly all of the BA.1-specific NAbs showed poor cross-reactivity against other Omicron subvariants (Fig. 6d). Specifically, most antibodies in the F3^Omi^ and A^Omi^ groups are evaded by BA.2 subvariants and BA.3, possibly because of D405N, and antibodies in B^Omi^ are evaded by BA.4 because of F486V. Binding of some group D^Omi^ antibodies may be affected by S446G but were not detected by DMS; these antibodies were evaded by BA.2 subvariants and BA.4 (Fig. 6g). To further validate the results obtained by DMS, we constructed pseudoviruses based on BA.1 harbouring the reverting spike mutations N417K, K440N, S446G, K478T, A484E, R498Q, Y501N and H505Y, as well as BA.1 spike(D405N) and BA.1 spike(R408S). Virus expressing BA.1 spike(D405N) did not produce sufficient titres for further experiments despite multiple attempts. We therefore used BA.2 spike(N405D) instead. We found that the N417K, R498Q, Y501N and H505Y reversions indeed led to evasion of most A^Omi^ and F3^Omi^ group antibodies, consistent with results from DMS (Fig. 6g). K484E and K478T are the major escaping mutants responsible for the poor breadth of B^Omi^ NAbs (Fig. 6d). S446G caused a small subset of D^Omi^ antibodies to lose neutralization potency, whereas R498Q and K440N resulted in the majority of D^Omi^ NAbs not binding to WT RBD. Of note, expression of BA.1 spike(R408S) did not reduce neutralization by BA.1-specific NAbs, whereas BA.2 spike(N405D) restored the neutralization potency of A^Omi^ and F3^Omi^ group antibodies against BA.2, indicating that D405N is the determinant of their poor cross-reactivity among BA.2, BA.3, BA.4 and BA.5 sublineages (Fig. 6d,g). These BA.1-specific NAbs displayed different heavy chain V gene usage compared to WT-reactive antibodies in the corresponding epitope group. Specifically, antibodies in A^Omi^ and B^Omi^ groups did not show significant convergence. IGHV3-53 and IGHV3-66 contributes only to a small subset of group A^Omi^ antibodies. Instead, group D^Omi^ antibodies were dominated by IGHV2-70 andIGHV5-51, whereas F^Omi^ was dominated by IGHV4-59 (Extended Data Fig. 10). These three heavy chain V genes also appeared in WT-reactive antibodies, but were relatively rare and did not show significant epitope enrichment (Extended Data Fig. 6a,b).

Here we have shown that Omicron is continuously evolving under immune pressure, and rationalized the appearance of R346K (BA.1.1), L452 substitutions and the F486V mutation, which have enabled increased immune evasion. In contrast to when Omicron first appeared, new Omicron sublineages are able to target the humoral immunity induced by Omicron itself, such as by post-vaccination Omicron infection. Omicron breakthrough infections mainly recall WT-induced memory B cells^40,41^, which in turn narrow the diversity of elicited antibodies and may further drive the appearance of future mutants. These phenomena pose a challenge to the current herd immunity established through WT-based vaccination and infection by BA.1 and BA.2 variants, which is concordant with recent observations^42^. Similarly, these results also suggest that Omicron BA.1-based vaccine may not be the ideal antigen for inducing broad-spectrum protection against emerging Omicron sublineages.

By combining high-throughput single-cell sequencing and high-throughput yeast display-based DMS, we have demonstrated the ability to decipher the complicated humoral immune repertoire elicited by Omicron infection and the underlying immune-evasion mechanism of L452 and F486 mutations. The ability to dissect the entire humoral immunity into distinct antibody epitope groups greatly increases the resolution of antibody and mutational escape research. The antibodies in each epitope group show highly concordant attributes and features, which will facilitate the investigation of the immune-evasion mechanism of circulating variants. This work and the comprehensive data that we have generated here will inform the development of broad-spectrum sarbecovirus vaccines and therapeutic antibodies.

## Methods

### Plasma and PBMC isolation

Blood samples were obtained from 40 volunteers who had received 3 doses of CoronaVac, 39 individuals who had received 2 doses of CoronaVac and 1 booster dose of ZF2001, 54 individuals who had recovered from BA.1 infection who had previously received 3 doses of CoronaVac^43,44^, and 30 individuals who had recovered from SARS who had received 2 doses of CoronaVac and 1 dose of ZF2001. The volunteers’ blood samples were obtained four weeks after the booster shot or four weeks after discharge from the hospital following BA.1 infection.COVID-19 disease severity was defined as asymptomatic, mild, moderate, severe or critical according to the *WHO Living Guidance for Clinical Management of COVID-19*^45^. Relevant experiments with plasma from SARS convalescents and SARS-CoV-2 vaccinees were approved by the Beijing Ditan Hospital Capital Medical University (ethics committee archiving no. LL-2021-024-02), the Tianjin Municipal Health Commission, and the ethics committee of Tianjin First Central Hospital (ethics committee archiving no. 2022N045KY). Written informed consent was obtained from each participant in accordance with the Declaration of Helsinki. All participants provided written informed consent for the collection of information, storage and usage of their clinical samples for research purpose, and publication of data generated from this study.

Whole blood samples were mixed and subjected to Ficoll (Cytiva, 17-1440-03) gradient centrifugation after 1:1 dilution in PBS+2% FBS to isolate plasma and PBMCs. After centrifugation, plasma was collected from upper layer and cells were collected at the interface. PBMCs were further prepared by centrifugation, red blood cells lysis (Invitrogen eBioscience 1X RBC Lysis Buffer, 00-4333-57) and washing steps. Samples were stored in FBS (Gibco) with 10% DMSO (Sigma) in liquid nitrogen if not used for downstream process immediately. Cryopreserved PBMCs were thawed in DPBS + 2% FBS (Stemcell, 07905).

### Ethics statement

This study was approved by the Ethics Committee of Beijing Ditan Hospital affiliated to Capital Medical University (Ethics committee archiving No. LL-2021-024-02), the Tianjin Municipal Health Commission, and the Ethics Committee of Tianjin First Central Hospital (Ethics committee archiving No. 2022N045KY). Informed consent was obtained from all human research participants.

### Antibody isolation and recombinant production

SARS-CoV-1 and SARS-CoV-2 RBD cross-binding memory B cells were isolated from PBMC of SARS convalescents who had received SARS-CoV-2 vaccine and BA.1-infected convalescents who had been vaccinated against COVID-19 prior to infection. In brief, CD19^+^ B cells were isolated from PBMCs with EasySep Human CD19 Positive Selection Kit II (STEMCELL, 17854). Every 10^6^ B cells in 100 μl were then stained with 2.5 μl FITC anti-human CD19 antibody (BioLegend, 392508), 2.5 μl FITC anti-human CD20 antibody (BioLegend, 302304), 3.5 μl Brilliant Violet 421 anti-human CD27 antibody (BioLegend, 302824), 3 μl PE/Cyanine7 anti-human IgM antibody (BioLegend, 314532), 0.21 μg biotinylated Ovalbumin (Sino Biological) conjugated with Brilliant Violet 605 Streptavidin (BioLegend, 405229), 0.13 μg SARS-CoV-1 biotinylated RBD protein (His and AVI Tag) (Sino Biological, 40634-V27H-B) conjugated with PE-streptavidin (BioLegend, 405204), 0.13 μg SARS-CoV-2 biotinylated RBD protein (His and AVI Tag) (Sino Biological, 40592-V27H-B) conjugated with APC-streptavidin (BioLegend, 405207), and 5 μl 7-AAD (Invitrogen, 00-6993-50). 7-AAD^−^CD19/CD20^+^CD27^+^IgM^−^OVA^−^ SARS-COV-1 RBD^+^ and SARS-CoV-2 RBD^+^ cells were sorted with a MoFlo Astrios EQ Cell Sorter (Beckman Coulter).

SARS-CoV-2 BA.1 RBD-binding memory B cells were isolated from BA.1-infected convalescents who received SARS-CoV-2. In brief, CD19^+^ B cells were isolated with EasySep Human CD19 Positive Selection Kit II. Every 10^6^ B cells in 100 μl solution were then stained with 3 μl FITC anti-human CD20 antibody (BioLegend, 302304), 3.5 μl Brilliant Violet 421 anti-human CD27 antibody (BioLegend, 302824), 2 μl PE/Cyanine7 anti-human IgM antibody (BioLegend, 314532), 2 μl PE/Cyanine7 anti-human IgD antibody (BioLegend, 348210), 0.13 μg biotinylated SARS-CoV-2 BA.1 protein (His and AVI Tag) (Sino Biological, 40592-V49H7-B) conjugated with PE-streptavidin or APC-streptavidin (TotalSeq-C0971 Streptavidin, BioLegend, 405271 and TotalSeq-C0972 Streptavidin, BioLegend, 405273), 0.13 μg SARS-CoV-2 WT biotinylated RBD protein (His and AVI Tag) conjugated with Brilliant Violet 605 Streptavidin and TotalSeq-C0973 Streptavidin (BioLegend, 405275) and TotalSeq-C0974 Streptavidin(BioLegend, 405277), 0.21 μg biotinylated Ovalbumin conjugated with TotalSeq-C0975 Streptavidin (BioLegend, 405279) and 5 μl 7-AAD (Invitrogen, 00-6993-50). 7-AAD^−^CD20^+^CD27^+^IgM^−^IgD^−^ SARS-CoV-2 BA.1 RBD^+^ cells were sorted with a MoFlo Astrios EQ Cell Sorter. FACS data were analysed using FlowJo v10.8 (BD Biosciences).

Sorted B cells were then processed with Chromium Next GEM Single Cell V(D)J Reagent Kits v1.1 following the manufacturer’s user guide (10x Genomics, CG000208). In brief, sorted cells were resuspended in PBS after centrifugation. Gel beads-in-emulsion (GEMs) were obtained with 10X Chromium controller and then subjected to reverse transcription. After GEM-RT clean up, reverse transcription products were subject to preamplification. After amplification and purification with SPRIselect Reagent Kit (Beckman Coulter, B23318) of reverse transcription products, BCR sequences (paired V(D)J) were enriched with 10X BCR primers. After library preparation, libraries were sequenced by Novaseq 6000 platform running Novaseq 6000 S4 Reagent Kit v1.5300 cycles (Illumina, 20028312) or NovaSeq XP 4-Lane Kit v1.5 (Illumina, 20043131).

### B cell RNA and feature barcode data analysis

Using Cell Ranger (v6.1.1) pipeline, the mRNA fastq reads were processed and aligned to the human GRCh38 genome for gene expression profile. Genes expressed in fewer than 10 cells and cells expressing fewer than 100 genes or high-level mitochondria genes were removed to filter out low-quality data. Raw counts were normalized and scaled with Seurat^46^ (v 4.0.3), while principal components analysis and uniform manifold approximation and projection were performed for cluster and visualization. Cell types were identified using SingleR^47^ (v1.6.1) with Monaco human immune data^48^. Feature barcode reads were also counted by Cell Ranger (v6.1.1) as antibody capture library, and a cell was considered to bind the corresponding antigen of dominant feature barcodes (>25% in this cell).

### Antibody sequence analysis

The antibody sequences obtained from 10X Genomics V(D)J sequencing were aligned to GRCh38 reference and assembled as immunoglobulin contigs by the Cell Ranger (v6.1.1) pipeline. Non-productive contigs and B cells that had multiple heavy chain or light chain contigs were filtered out of the analysis. V(D)J gene annotation was performed using NCBI IgBlast (v1.17.1) with the IMGT reference. Mutations on V(D)J nucleotide sequences were calculated by using the igpipeline, which compared the sequences to the closest germline genes and counted the number of different nucleotides. For antibodies from public sources whose original sequencing nucleotide sequences were not all accessible, the antibody amino acid sequences were annotated by IMGT/DomainGapAlign^49^ (v4.10.2) with default parameters. V–J pairs were visualized with the R package circlize (v0.4.10).

### DMS library construction

DMS libraries were constructed as previously described^3^. In brief, SARS-CoV-2 RBD mutant libraries were constructed from Wuhan-Hu-1 RBD sequence (GenBank: MN908947, residues N331–T531), and Omicron RBD mutant libraries were created in a similar way based on Wuhan-Hu-1 RBD sequence with the addition of G339D, S371L, S373P, S375F, K417N, N440K, G446S, S477N, T478K, E484A, Q493R, G496S, Q498R, N501Y and Y505H mutations. Duplicate libraries were produced independently, theoretically containing 3,819 possible amino acid mutations. Each RBD mutant was barcoded with a unique 26-nucleotide (N26) sequence and Pacbio sequencing was used to identify the correspondence of RBD mutant and N26 barcode. After mutant library transformation, ACE2 binders were enriched for downstream mutation profile experiments.

### High-throughput antibody-escape mutation profiling

The magnetic-activated cell sorting-based antibody-escape mutation profiling system^3,17^ was used to characterize mutation escape profile for NAbs. In brief, ACE2-binding mutants were induced overnight for RBD expression and washed followed with two rounds of Protein A antibody-based negative selection and MYC tag-based positive selection to enrich RBD-expressing cells. Protein A antibody-conjugated products were prepared following the protocol for Dynabeads Protein A (Thermo Fisher, 10008D) and incubated with induced yeast libraries at room temperature for 30 min with shaking. MYC tag-based positive selection was performed according to the manufacturer’s instructions (Thermo Fisher, 88843).

After three rounds of sequential cell sorting, the obtained cells were recovered overnight. Plasmids were extracted from pre- and post-sort yeast populations by 96-Well Plate Yeast Plasmid Preps Kit (Coolaber, PE053). The extracted plasmids were then used to amplify N26 barcode sequences by PCR. The final PCR products were purified with 1X AMPure XP magnetic beads (Beckman Coulter, A63882) and submitted to 75bp single-end sequencing at Illumina Nextseq 500 platform.

### Processing of DMS data

Single-end Illumina sequencing reads were processed as previously described. In brief, reads were trimmed to 16 or 26 bp and aligned to the reference barcode-variant dictionary with dms_variants package (v0.8.9). Escape scores of variants were calculated as *F* × (*n*_X,ab_/*N*_ab_)/(*n*_X,ref_/*N*_ref_), where *n*_X,ab_ and *n*_X,ref_ is the number of reads representing variant X, and *N*_ab_ and *N*_ref_ are the total number of valid reads in antibody-selected (ab) and reference (ref) library, respectively. *F* is a scale factor defined as the 99th percentiles of escape fraction ratios. Variants detected by less than six reads in the reference library were removed to avoid sampling noise. Variants containing mutations with ACE2 binding below −2.35 or RBD expression below −1 were removed as well, according to data previously reported. For BA.1 RBD-based libraries, due to the lack of corresponding ACE2-binding and RBD expression data, we used the RBD expression of Beta RBD-based DMS as filter instead^50^, and did not perform the ACE2-binding filter. Mutations on residues that use different amino acids in Beta and BA.1 were not filtered, except R493P, S496P, R498P, H505P and all mutations on F375, which were excluded in the analysis owing to low expression. Finally, global epistasis models were built using dms_variants package to estimate mutation escape scores. For most antibodies, at least two independent assays were conducted and single mutation escape scores were averaged across all experiments that pass quality control.

### Antibody clustering and visualization

Site total escape scores, defined as the sum of escape scores of all mutations at a particular site on RBD, were used to evaluate the impact of mutations on each site for each antibody. Each of these scores is considered as a feature of a certain antibody and used to construct a feature matrix *A*_*N*×*M*_ for downstream analysis, where *N* is the number of antibodies and *M* is the number of features (valid sites). Informative sites were selected using sklearn.feature_selection.VarianceThreshold of scikit-learn Python package (v0.24.2) with the variance threshold as 0.1. Then, the selected features were L2-normalized across antibodies using sklearn.preprocessing.normalize. The resulting matrix is referred as *A*′_*N*×*M*__′_, where *M*′ is the number of selected features. The dissimilarity of two antibodies *i*, *j* is defined as 1 − Corr(*A*′_*i*_,*A*′_*j*_), where Corr(**x**,**y**) is the Pearson’s correlation coefficient of vectors **x** and **y**. We used sklearn.manifold.MDS to reduce the number of features from *M*′ to *D* = 20 with multidimensional scaling under the above metric. Antibodies are clustered into 12 epitope groups using sklearn.cluster.KMeans of scikit-learn in the resulting *D*-dimensional feature space. Finally, these *D*-dimensional representations of antibodies were further embedded into two-dimensional space for visualization with *t*-SNE using sklearn.manifold.TSNE of scikit-learn. For the 102 BA.1-specific antibodies that were assayed with BA.1 RBD-based yeast display library, the 20-dimensional embedding was generated using  multidimensional scaling (MDS) with the DMS profile of all 1,640 antibodies, but clustering and *t*-SNE were conducted independently. To project these antibodies onto the *t*-SNE space of 1,538 antibodies assayed by WT RBD-based DMS, we calculated the pairwise Euclidean distance between 102 antibodies using BA.1 RBD-based DMS and 1,538 antibodies using WT RBD-based DMS in the 20-dimensional MDS space. The position of each BA.1-specific antibody in the original *t*-SNE space is defined as the average position of its ten nearest antibodies using WT RBD-based DMS. All *t*-SNE plots were generated by R package ggplot2 (v3.3.3).

### Pseudovirus-neutralization assay

SARS-CoV-2 spike (GenBank: MN908947), Pangolin-GD spike (GISAID: EPI_ISL_410721), RaTG13 spike (GISAID: EPI_ISL_402131), SARS-CoV-1 spike (GenBank: AY278491), Omicron BA.1 spike (A67V, H69del, V70del, T95I, G142D, V143del, Y144del, Y145del, N211del, L212I, ins214EPE, G339D, S371L, S373P, S375F, K417N, N440K, G446S, S477N, T478K, E484A, Q493R, G496S, Q498R, N501Y, Y505H, T547K, D614G, H655Y, N679K, P681H, N764K, D796Y, N856K, Q954H, N969K, L981F), BA.2 spike (GISAID: EPI_ISL_7580387, T19I, L24S, del25-27, G142D, V213G, G339D, S371F, S373P, S375F, T376A, D405N, R408S, K417N, N440K, G446S, S477N, T478K, E484A, Q493R, Q498R, N501Y, Y505H, D614G, H655Y, N679K, P681H, N764K, D796Y, Q954H, N969K), BA.1.1 spike (BA.1+R346K), BA.3 spike (A67V, del69-70, T95I, G142D, V143del, Y144del, Y145del, N211del, L212I, G339D, S371F, S373P, S375F, D405N, K417N, N440K, G446S, S477N, T478K, E484A, Q493R, Q498R, N501Y, Y505H, D614G, H655Y, N679K, P681H, N764K, D796Y, Q954H, N969K), BA.2.12.1 spike (BA.2+L452Q+S704L), BA.2.13 spike (BA.2+L452M) and BA.4 spike (T19I, L24S, del25-27, del69-70, G142D, V213G, G339D, S371F, S373P, S375F, T376A, D405N, R408S, K417N, N440K, G446S, L452R, S477N, T478K, E484A, F486V, Q498R, N501Y, Y505H, D614G, H655Y, N679K, P681H, N764K, D796Y, Q954H, N969K) plasmids were constructed using the pcDNA3.1 vector. G*ΔG-VSV virus (VSV G pseudotyped virus, Kerafast) was used to infect 293T cells (American Type Culture Collection (ATCC), CRL-3216), and spike protein-expressing plasmid was used for transfection at the same time. After culture, the supernatant containing pseudovirus was collected, filtered, aliquoted, and frozen at −80 °C for further use.

Pseudovirus detection of Pangolin-GD and RaTG13 was performed in 293T cells overexpressing human angiotensin-converting enzyme 2 (293T-hACE2 cells). Other pseudovirus-neutralization assays were performed using the Huh-7 cell line (Japanese Collection of Research Bioresources (JCRB), 0403).

Monoclonal antibodies or plasma were serially diluted (fivefold or threefold) in DMEM (Hyclone, SH30243.01) and mixed with pseudovirus in 96-well plates. After incubation at 5% CO_2_ and 37 °C for 1 h, digested Huh-7 cell (JCRB, 0403) or 293T-hACE2 cells (ATCC, CRL-3216) were seeded. After 24 h of culture, supernatant was discarded and d-luciferin reagent (PerkinElmer, 6066769) was added to react in the dark, and the luminescence value was detected using a microplate spectrophotometer (PerkinElmer, HH3400). IC_50_ was determined by a four-parameter logistic regression model using PRISM (version 9.0.1).

### ELISA

To detect the broad-spectrum binding of the antibodies among Sarbecovirus, we used a panel of 20 synthesized sarbecovirus RBDs (Sino Biological Technology) (Supplementary Table 3). According to the sequence of 20 RBDs, a set of nested primers was designed. The coding sequences were obtained by the overlap PCR with a 6× His tag sequence to facilitate protein purification. The purified PCR products were ligated to the secretory expression vector pCMV3 with CMV promoter, and then transformed into *Escherichia coli* XL1-blue competent cells. Monoclones with correct transformation were cultured and expanded, and plasmids were extracted. Healthy HEK293F cells were passaged into a new cell culture and grown in suspension at 37 °C, 120 RPM, 8% CO2 to logarithmic growth phase and transfected with the recombinant constructs by using liposomal vesicles as DNA carrier. After transfection, the cell cultures were followed to assess the kinetics of cell growth and viability for 7 days. The cell expression supernatant was collected, and after centrifugation, passed through a Ni column for affinity purification. The molecular size and purity of eluted protein was confirmed by SDS–PAGE. Production lot numbers and concentration information of the 20 sarbecovirus proteins are shown in Supplementary Table 4. WT RBD used here was SARS-CoV-2 (2019-nCoV) Spike RBD-His Recombinant Protein (Sino Biological, 40592-V08H).

A panel of 21 sarbecovirus RBDs (Supplementary Table 3) in PBS was pre-coated onto ELISA plates (NEST, 514201) at 4 °C overnight. The plates were washed and blocked. Then 1 μg ml^−1^ purified antibodies or serially diluted antibodies were added and incubated at room temperature for 20 min. Next, Peroxidase-conjugated AffiniPure Goat Anti-Human IgG (H+L) (JACKSON, 109-035-003) was applied and incubated at room temperature for 15 min. Tetramethylbenzidine (TMB) (Solarbio, 54827-17-7) was added onto the plates. The reaction was terminated with 2 M H_2_SO_4_ after 10 min incubation. Absorbance was measured at 450 nm using Ensight Multimode Plate Reader (PerkinElmer, HH3400). ELISA *A*_450_ measurements at different antibody concentrations for a particular antibody–antigen pair were fit to the model *y* = *Ac*^*n*^/(*c*^*n*^ + *E*^*n*^) using the R package mosaic (v1.8.3), where *y* is the *A*_450_ value and *c* is the corresponding antibody concentration. *A*, *E* and *n* are parameters, where *E* is the desired EC_50_ value for the specific antibody and antigen.

### Antibody and ACE2 competition for RBD

Omicron RBD (Sino Biological, 40592-V08H121) protein in PBS was immobilized on the ELISA plates at 4 °C overnight. The coating solution was removed and washed 3 times with PBST and the plates were then blocked for 2 h. After blocking, the plates were washed 5 times, and the mixture of ACE2–biotin (Sino Biological, 10108-H27B-B) and serially diluted competitor antibodies was added followed by 30 min incubation at room temperature. Peroxidase-conjugated Streptavidin (Jackson ImmunoResearch, 016-030-084) was added into each well for another 20 min incubation at room temperature. After washing the plates five times, TMB (Solarbio, 54827-17-7) was added into each well. After 10 min, the reaction was terminated with 2 M H_2_SO_4_. Absorbancewas measured at 450 nm using Ensight Multimode Plate Reader (PerkinElmer, HH3400). The ACE2 competition coefficient was calculated as (*B* − *A*)/*B*, where *B* is the *A*_450_ value with 0.3 μg ml^−1^ antibody and *A* is the *A*_450_ value with 6 μg ml^−1^ antibody.

### Biolayer interferometry

Biolayer interferometry assays were performed on Octet RED 384 Protein Analysis System (Fortebio) according to the manufacturer’s instructions. To measure the binding affinities, monoclonal antibodies were immobilized onto Protein A biosensors (Fortebio) and the fourfold serial dilutions of Omicron S-trimer (BA.1 and BA.2) in PBS were used as analytes. Data were collected with Octet Acquisition 9.0 (Fortebio) and analysed by Octet Analysis 9.0 (Fortebio) and Octet Analysis Studio 12.2 (Fortebio).

### S-trimer thermal stability assay

The thermal stability assay was performed to detect the exposed hydrophobic residues with an MX3005 qPCR instrument (Agilent) with SYPRO Red (Invitrogen) as fluorescent probes. We set up 25 μl reaction system (pH 8.0) containing 5 μg of target protein (S-trimer of Omicron lineage), 1000× SYPRO Red, and ramped up the temperature from 25 °C to 99 °C. Fluorescence was recorded in triplicate at an interval of 1 °C.

### Surface plasmon resonance

Human ACE2 was immobilized onto CM5 sensor chips using a Biacore 8K (GE Healthcare). Serial dilutions of purified S-trimer or RBD of Omicron lineages were injected, ranging in concentrations from 100 to 6.25 nM. The response units were recorded at room temperature using BIAcore 8K Evaluation Software (v3.0.12.15655; GE Healthcare), and the resulting data were fitted to a 1:1 binding model using BIAcore 8K Evaluation Software (v3.0.12.15655; GE Healthcare).

### Protein expression and purification for cryo-EM study

The S6P expression construct encoding the SARS-CoV-2 spike ectodomain (residues 1–1208) with six stabilizing Pro substitutions (F817P, A892P, A899P, A942P, K986P and V987P) and a GSAS substitution for the furin cleavage site (residues 682–685) was previously described^15^. The Delta-specific mutations (T19R, G142D, 156del, 157del, R158G, L452R, T478K, D614G, P681R, D950N) were introduced into this construct using site-directed mutagenesis. The S6P expression construct containing the Omicron BA.1 mutations (A67V, H69del, V70del, T95I, G142D, V143del, Y144del, Y145del, N211del, L212I, ins214EPE, G339D, S371L, S373P, S375F, K417N, N440K, G446S, S477N, T478K, E484A, Q493R, G496S, Q498R, N501Y, Y505H, T547K, D614G, H655Y, N679K, P681H, N764K, D796Y, N856K, Q954H, N969K, L981F) were assembled from three synthesized DNA fragments. The S6P expression construct containing the Omicron BA.2 mutations (T19I, L24S, del25-27, G142D, V213G, G339D, S371F, S373P, S375F, T376A, D405N, R408S, K417N, N440K, G446S, S477N, T478K, E484A, Q493R, Q498R, N501Y, Y505H, D614G, H655Y, N679K, P681H, N764K, D796Y, Q954H, N969K) were assembled from three synthesized DNA fragments. The S6P expression construct containing the Omicron BA.4/BA.5 mutations (T19I, L24S, del25-27, del69-70, G142D, V213G, G339D, S371F, S373P, S375F, T376A, D405N, R408S, K417N, N440K, G446S, L452R, S477N, T478K, E484A, F486V, Q498R, N501Y, Y505H, D614G, H655Y, N658S, N679K, P681H, N764K, D796Y, Q954H, N969K) were assembled from three synthesized DNA fragments^51^. For protein production, these expression plasmids, as well as the plasmids encoding the antigen-binding fragments (Fabs) of the antibodies described here, were transfected into the HEK293F cells using polyethylenimine (Polysciences). The conditioned media were collected and concentrated using a Hydrosart ultrafilter (Sartorius), and exchanged into the binding buffer (25 mM Tris, pH 8.0, and 200 mM NaCl). Protein purifications were performed using the Ni-NTA affinity method, followed by gel filtration chromatographies using either a Superose 6 increase column (for the spike proteins) or a Superose 200 increase column (for the Fabs). The final buffer used for all proteins is 20 mM HEPES, pH 7.2, and 150 mM NaCl.

### Cryo-EM data collection, processing and structure building

Samples for cryo-EM study were prepared essentially as described^15,52^ (Supplementary Table 4). All EM grids were evacuated for 2 min and glow-discharged for 30 s using a plasma cleaner (Harrick PDC-32G-2). Four microliters of spike protein (0.8 mg ml^−1^) was mixed with the same volume of Fabs (1 mg ml^−1^ each), and the mixture was immediately applied to glow-discharged holy-carbon gold grids (Quantifoil, R1.2/1.3) in an FEI Vitrobot IV (4 °C and 100% humidity). Data collection was performed using either a Titan Krios G3 equipped with a K3 direct detection camera, or a Titan Krios G2 with a K2 camera, both operating at 300 kV. Data processing was carried out using cryoSPARC (v3.2.1)^53^. After 2D classification, particles with good qualities were selected for global 3D reconstruction and then subjected to homogeneous refinement. To improve the density surrounding the RBD–Fab region, UCSF Chimera (v1.16)^54^ and Relion (v3.1)^55^ were used to generate the masks, and local refinement was then performed using cryoSPARC (v3.2.1). Coot (v0.8.9.2)^56^ and Phenix (v1.20)^57^ were used for structural modelling and refinement. Figures were prepared using USCF ChimeraX (v1.3)^58^ and Pymol (v2.4.0, Schrödinger, LLC.).

### Molecular dynamics simulation

Models of the RBD from BA.1, BA.2, BA.3, BA.2.13, BA.2.12.1 and BA.4 in complex with ACE2 were firstly referred to the cryo-EM structure of BA.1–hACE2 (PDB code: 7WGB) and then checked with the WHAT IF web interface (https://swift.cmbi.umcn.nl/) to remove atomic clashes. After that, the structures were simulated with GROMACS-2021^59^. In brief, the OPLS force field with TIP3P water model was selected to prepare the dynamic system. After that Na^+^ and Cl^−^ ions were added into the system to make the system electrically neutral. Then, energy minimization using the steepest descent algorithm was carried out until the maximum force of 1,000 kJ mol^−1^ was achieved. NVT ensemb1e via the Nose-Hoover method at 300 K and NPT ensemble at 1 bar with the Parinello-Rahman algorithm were employed successively to make the temperature and the pressure equilibrated, respectively. Finally, molecular dynamics production runs of 10 ns were performed with random initial velocities and periodic boundary conditions. The non-bonded interactions were treated using Verlet cut-off scheme, while the long-range electrostatic interactions were treated using particle mesh Ewald method^59^. The short-range electrostatic and van der Waals interactions were calculated with a cut-off of 12 Å. All six models were simulated in the same protocol.

### Reporting summary

Further information on research design is available in the Nature Research Reporting Summary linked to this paper.

## Online content

Any methods, additional references, Nature Research reporting summaries, source data, extended data, supplementary information, acknowledgements, peer review information; details of author contributions and competing interests; and statements of data and code availability are available at 10.1038/s41586-022-04980-y.

## Supplementary information

Reporting Summary

Supplementary Table 1 Summarized information of SARS-CoV-2 vaccinated individuals, BA.1 convalescents and SARS convalescents involved in the study.

Supplementary Table 2 Summarized information and experiment results of 1640 SARS-CoV-2 RBD antibodies involved in this study, including their sources, epitope groups, pseudovirus neutralizing IC50 and ELISA OD450 for sarbecovirus, ACE2 competition levels, and heavy/light chain sequences.

Supplementary Table 3 Accession numbers of sequences of sarbecovirus RBD used in the study.

Supplementary Table 4 Reports of detailed parameters of the reconstructed Cryo-EM structures.

## Data Availability

Processed mutation escape scores can be downloaded from https://github.com/jianfcpku/SARS-CoV-2-RBD-DMS-broad. Raw Illumina and PacBio sequencing data are available on NCBI Sequence Read Archive BioProject PRJNA804413. We used vdj_GRCh38_alts_ensembl-5.0.0 as the reference for V(D)J alignment, which can be obtained from https://support.10xgenomics.com/single-cell-vdj/software/downloads/latest. IMGT/DomainGapAlign is based on the built-in lastest IMGT antibody database, and we set the ‘Species’ parameter as ‘Homo sapiens’ and kept the others at the default settings. Public DMS datasets involved in the study from literature can be downloaded from https://media.githubusercontent.com/media/jbloomlab/ SARS2_RBD_Ab_escape_maps/main/ processed_data/escape_data.csv. Cryo-EM density maps have been deposited in the Electron Microscopy Data Bank with accession codes EMD-33210, EMD-33211, EMD-33212, EMD-33213, EMD-33323, EMD-33324, EMD-33325, EMD-32732, EMD-32738, EMD-32734, EMD-32718 and EMD-33019. Structural coordinates have been deposited in the Protein Data Bank with accession codes 7XIW, 7XIX, 7XIY, 7XIZ, 7XNQ, 7XNR, 7XNS, 7WRL, 7WRZ, 7WRO, 7WR8 and 7X6A.

## Code availability

Python and R scripts for analysing escaping mutation profile data and reproducing figures in this manuscript are available at https://github.com/jianfcpku/SARS-CoV-2-RBD-DMS-broad.
