# Supplementary material for: BA.2.12.1, BA.4 and BA.5 escape antibodies elicited by Omicron infection
Source: Nature. 2022 Jun 17;608(7923):593–602. doi: 10.1038/s41586-022-04980-y (PMC9385493; doi:10.1038/s41586-022-04980-y)
Supplement: Supplementary file 1 — Reporting Summary [file 41586_2022_4980_MOESM1_ESM.pdf]

nature portfolio

Corresponding author(s): Yunlong Cao, Zhongyang Shen, Xiangxi Wang, Youchun Wang, Junyu Xiao, Xiaoliang Sunney Xie

Last updated by author(s): Jun 10, 2022

Reporting Summary

Nature Portfolio wishes to improve the reproducibility of the work that we publish. This form provides structure for consistency and transparency in reporting. For further information on Nature Portfolio policies, see our Editorial Policies and the Editorial Policy Checklist.

Statistics

For all statistical analyses, confirm that the following items are present in the figure legend, table legend, main text, or Methods section.

n/a	Confirmed	
☐	☒	The exact sample size (n) for each experimental group/condition, given as a discrete number and unit of measurement
☐	☒	A statement on whether measurements were taken from distinct samples or whether the same sample was measured repeatedly
☐	☒	The statistical test(s) used AND whether they are one- or two-sided<br>*Only common tests should be described solely by name; describe more complex techniques in the Methods section.*
☒	☐	A description of all covariates tested
☒	☐	A description of any assumptions or corrections, such as tests of normality and adjustment for multiple comparisons
☐	☒	A full description of the statistical parameters including central tendency (e.g. means) or other basic estimates (e.g. regression coefficient) AND variation (e.g. standard deviation) or associated estimates of uncertainty (e.g. confidence intervals)
☐	☒	For null hypothesis testing, the test statistic (e.g. F, t, r) with confidence intervals, effect sizes, degrees of freedom and P value noted<br>*Give P values as exact values whenever suitable.*
☒	☐	For Bayesian analysis, information on the choice of priors and Markov chain Monte Carlo settings
☒	☐	For hierarchical and complex designs, identification of the appropriate level for tests and full reporting of outcomes
☒	☐	Estimates of effect sizes (e.g. Cohen's d, Pearson's r), indicating how they were calculated

Our web collection on statistics for biologists contains articles on many of the points above.

Software and code

Policy information about availability of computer code

Data collection	Pseudovirus neutralization and ELISA data were collected by microplate spectrophotometer (PerkinElmer, HH3400).<br>Biolayer interferometry data were collected with Octet Acquisition 9.0 (Fortebio).<br>FACS data was collected by Summit 6.0 (Beckman Coulter).<br>SPR data were obtained with BIAcore 8K Evaluation Software (v3.0.12.15655)<br>Cryo-EM data collection was performed using either a Titan Krios G3 equipped with a K3 direct detection camera, or a Titan Krios G2 with a K2 camera.

Data analysis

Neutralization assays data were analyzed using PRISM (versions 9.0.1) as described in Methods.
BLI data analyses were done by Octet Analysis 9.0 (Fortebio) and Octet Analysis Studio 12.2 (Fortebio).
SPR data were fitted with Biacore 8K Evaluation Software (v3.0.12.15655).
FACS data were analyzed by FlowJo 10.8.
V(D)J sequence data were analyzed using Cell Ranger (v6.1.1) and IMGT/DomainGapAlign (v4.10.2), and R packages Seurat (v4.0.3), SingleR (v1.6.1) .
Illumina barcodes sequencing data from deep mutational scanning experiments were analyzed using custom scripts (https://github.com/jianfcpku/SARS-CoV-2-RBD-DMS-broad) and Python package dms_variants (v0.8.9).
Logo plots were generated by Python package logomaker (version 0.8) and R package ggseqlogo (version 0.1). For unsupervised clustering, we utilized Python package scikit-learn (version 0.24.2) to perform multidimensional scaling (MDS), k-means clustering and t-Distributed Stochastic Neighbor Embedding (t-SNE) embedding. 2D t-SNE plots are generated by ggplot2 (version 3.3.3).
Multiple sequence alignments of sarbecovirus RBD were generated using ClustalOmega (version 1.2.4).
Cryo-EM data processing was carried out using cryoSPARC (v3.2.1), UCSF Chimera (v1.16) and Relion (v3.1). MD-simulation based on cryo-EM structures was carried out using GROMACS-2021. Coot (v0.8.9.2) and Phenix (v1.20) were used for cryo-EM structural modeling and refinement. Structure figures were prepared using USCF ChimeraX (v1.3) and Pymol (v2.6.0a0).

For manuscripts utilizing custom algorithms or software that are central to the research but not yet described in published literature, software must be made available to editors and reviewers. We strongly encourage code deposition in a community repository (e.g. GitHub). See the Nature Portfolio guidelines for submitting code & software for further information.

Data

Policy information about availability of data

All manuscripts must include a data availability statement. This statement should provide the following information, where applicable:

- Accession codes, unique identifiers, or web links for publicly available datasets
- A description of any restrictions on data availability
- For clinical datasets or third party data, please ensure that the statement adheres to our policy

Processed mutation escape scores can be downloaded at https://github.com/jianfcpku/SARS-CoV-2-RBD-DMS-broad. Raw Illumina and PacBio sequencing data are available on NCBI Sequence Read Archive BioProject PRJNA804413. We used vdj_GRCh38_alts_ensembl-5.0.0 as the reference of V(D)J alignment, which can be obtained from https://support.10xgenomics.com/single-cell-vdj/software/downloads/latest. IMGT/DomainGapAlign is based on the built-in lastest IMGT antibody database, and we let the "Species" parameter as "Homo sapiens" while kept the others as default. Public deep mutational scanning datasets involved in the study from literature could be downloaded at https://media.githubusercontent.com/media/jbloomlab/SARS2_RBD_Ab_escape_maps/main/processed_data/escape_data.csv. Public structures involved in this manuscript were downloaded from Protein Data Bank with accession codes 6M0J, 7K8V, 7MMO, 7EY0, 7DX4, 7M7W, 7JW0, 7WPB, 7WGB.
Cryo-EM density maps have been deposited in the Electron Microscopy Data Bank with accession codes EMD-33210, EMD-33211, EMD-33212, EMD-33213, EMD-33323, EMD-33324, EMD-33325, EMD-32732, EMD-32738, EMD-32734, EMD-32718, and EMD-33019, respectively. Structural coordinates have been deposited in the Protein Data Bank with accession codes 7XIW, 7XIX, 7XIY, 7XIZ, 7XNQ, 7XNR, 7XNS, 7WRL, 7WRZ, 7WRO, 7WR8 and 7X6A.

Field-specific reporting

Please select the one below that is the best fit for your research. If you are not sure, read the appropriate sections before making your selection.

☒ Life sciences ☐ Behavioural & social sciences ☐ Ecological, evolutionary & environmental sciences

For a reference copy of the document with all sections, see nature.com/documents/nr-reporting-summary-flat.pdf

Life sciences study design

All studies must disclose on these points even when the disclosure is negative.

Sample size

A total of 1640 antibodies were characterized in the manuscript. No sample size calculation was performed. The sample size of this study was chosen to obtain sufficient antibodies in each epitope group.
Plasma samples were obtained from 40 volunteers who received 3 doses of CoronaVac, 38 individuals who received 2 doses of CoronaVac and 1 booster dose of ZF2001, 50 BA.1 convalescents who had received 3 doses of CoronaVac before BA.1 infection, and 28 SARS convalescents who received 2 doses of CoronaVac and 1 dose of ZF2001. No sample size calculation was performed. All samples obtained were analyzed.

Data exclusions

457 antibodies were excluded from the study because of insufficient antibody or meaningless deep mutation screening results, which defined as no mutations scored two times of the median score.

Replication

Experimental assays were performed in biological duplicate according to or exceeding standards in the field.
Specifically, we perform MACS-based mutation screening using two independently synthesized mutant libraries. We conducted all neutralization and ELISA assays in biological replicates. SPR and BLI measurements are all performed in at least two biological replicates. All replicates for neutralization, ELISA, SPR and BLI assays are successful.

Randomization

Randomization was not required since we were applying a uniform set of measurements across the panel of monoclonal antibodies and plasma. As this is an observational study, randomization is not relevant.

Blinding

Blinding was not required since we were applying a uniform set of measurements across the panel of monoclonal antibodies and plasma. As this is an observational study, investigators were not blinded.

Reporting for specific materials, systems and methods

We require information from authors about some types of materials, experimental systems and methods used in many studies. Here, indicate whether each material, system or method listed is relevant to your study. If you are not sure if a list item applies to your research, read the appropriate section before selecting a response.

Materials & experimental systems

n/a	Involved in the study
☐	☒ Antibodies
☐	☒ Eukaryotic cell lines
☒	☐ Palaeontology and archaeology
☒	☐ Animals and other organisms
☐	☒ Human research participants
☒	☐ Clinical data
☒	☐ Dual use research of concern

Methods

n/a	Involved in the study
☒	☐ ChIP-seq
☐	☒ Flow cytometry
☒	☐ MRI-based neuroimaging

Antibodies

Antibodies used

ELISA: goat anti-human IgG(H+L)HRP (JACKSON, 109-035-003)
The enriched B cells were stained with FITC anti-human CD19 antibody (BioLegend, 392508), FITC anti-human CD20 antibody (BioLegend, 302304), Brilliant Violet 421 anti-human CD27 antibody (BioLegend, 302824), PE/Cyanine7 anti-human IgM antibody (BioLegend, 314532), PE/Cyanine7 anti-human IgD antibody(BioLegend, 348210), biotinylated SARS-CoV-2 BA.1 protein (His & AVI Tag) (SinoBiological, 40592-V49H7-B) conjugated with PE-streptavidin (BioLegend, 405204), APC-streptavidin (BioLegend, 405207), TotalSeq™-C0971 Streptavidin (BioLegend, 405271), and TotalSeq™-C0972 Streptavidin (BioLegend, 405273), SARS-CoV-2 biotinylated RBD protein (His & AVI Tag) conjugated with Brilliant Violet 605™ Streptavidin, TotalSeq™-C0973 Streptavidin(BioLegend, 405275), TotalSeq™-C0974 Streptavidin(BioLegend, 405277), biotinylated Ovalbumin conjugated with TotalSeq™-C0975 Streptavidin(BioLegend, 405279) and 7-AAD (Invitrogen, 00-6993-50).
All human antibodies were expressed using HEK293F cell lines with codon-optimized cDNA and human IgG1 constant regions in house. The detailed sequence could be found in Supplementary material.

Validation

In this manuscript, we tested 1640 human IgG1 antibodies. All antibodies were expressed using HEK293F cell lines with codon-optimized cDNA and human IgG1 constant regions. All antibodies' species and specificity to RBD were validated by ELISA. All antibodies neutralization ability was verified by VSV-based pseudotyped virus assays. Details and sequences for all SARS-CoV-2 antibodies evaluated in this study is included in Supplementary Table 2.
Reactivity and specificity of the primary antibodies listed above is based on the information on manufacturer's websites:
Goat anti-human IgG(H+L)HRP (JACKSON, 109-035-003): Based on immunoelectrophoresis and/or ELISA, the antibody reacts with whole molecule human IgG. It also reacts with the light chains of other human immunoglobulins. No antibody was detected against non-immunoglobulin serum proteins. The antibody may cross-react with immunoglobulins from other species.
FITC anti-human CD19 antibody was validated by successful staining and FC analysis according to the manufacturer's website https://www.biolegend.com/en-us/products/fitc-anti-human-cd19-antibody-16221 and previous publication: Riquelme SA, et al. 2020. Cell Metabolism. 31(6):1091-1106.e6.
FITC anti-human CD20 antibody was validated by successful staining and FC analysis according to the manufacturer's website https://www.biolegend.com/en-us/products/fitc-anti-human-cd20-antibody-558 and previous publication: Mishra A, et al. 2021. Cell. 184(13):3394-3409.e20.
Brilliant Violet 421 anti-human CD27 antibody was validated by successful staining and FC analysis according to the manufacturer's website https://www.biolegend.com/en-us/products/brilliant-violet-421-anti-human-cd27-antibody-7276 and previous publication: Dugan HL, et al. 2021. Immunity. 54(6):1290-1303.e7.
PE/Cyanine7 anti-human IgM antibody was validated by successful staining and FC analysis according to the manufacturer's website https://www.biolegend.com/en-us/products/pe-cyanine7-anti-human-igm-antibody-12467 and previous publication: Shehata L, et al. 2019. Nat Commun. 10:1126.
PE/Cyanine7 anti-human IgD antibody was validated by successful staining and FC analysis according to the manufacturer's website https://www.biolegend.com/en-us/products/pe-cyanine7-anti-human-igd-antibody-6996 and previous publication: Ahmed R et al. 2019. Cell. 177(6):1583-1599.

Eukaryotic cell lines

Policy information about cell lines

Cell line source(s)

HEK293F for antibody and sarbecovirus RBD production was received from ThermoFisher (R79007);
EBY100 (Yeast) was received from ATCC (ATCCMYA-4941);
Huh-7 for pseudovirus assays was received from Japanese Collection of Research Bioresources (JCRB 0403) ;
293T-hACE2 cells for pseudovirus assays was received from ATCC (CRL-3216) ;

Authentication

No authentication was performed beyond manufacturer standards;

Mycoplasma contamination

Not tested for mycoplasma contamination;

Commonly misidentified lines
(See ICLAC register)

No commonly misidentified cell lines were used in the study.

Human research participants

Policy information about studies involving human research participants

Population characteristics

Plasma samples were obtained from 40 volunteers who received 3 doses of CoronaVac, 38 individuals who received 2 doses of CoronaVac and 1 booster dose of ZF2001, 50 BA.1 convalescents who had received 3 doses of CoronaVac before BA.1 infection, and 28 SARS convalescents who received 2 doses of CoronaVac and 1 dose of ZF2001. For the CoronaVac-boosted cohort, individuals were vaccinated by 2 doses of CoronaVac and boosted with CoronaVac six months after the second dose. The blood samples were obtained 4 weeks after the booster dose (21-59 years old, 9/40 male, 31/40 female). For the ZF2001-boosted cohort, individuals were vaccinated by 2 doses of CoronaVac and boosted with ZF2001 six months after the second dose. The blood samples were obtained 4 weeks after the booster dose (20-57 years old, 11/38 male, 27/38 female). For the BA.1 breakthrough infection cohort, the blood samples were obtained 4 weeks after hospitalization discharge (23-63 years old, 28/50 male, 22/50 female, 24/50 diagnosed as mild COVID-19, 26/50 diagnosed as moderate COVID-19, all received 2 doses of CoronaVac and a CoronaVac booster dose six months after the second dose before infection). We presume all individuals were infected by BA.1 since these individuals were infected during the BA.1 wave in Tianjin, China in Jan 2022. A total of 430 patients were confirmed infected and no other lineages were detected beside BA.1 by sequencing in that wave. For the SARS convalescents, all individuals were infected by SARS-CoV-1 in 2003 in Beijing, China and vaccinated by 2 doses of CoronaVac and boosted with ZF2001 six months after the second dose. The blood samples were collected 4 weeks after the booster dose (39-76 years old, 10/28 male, 18/28 female).
Detailed population characteristics and vaccination profiles are described in Supplementary Table 1.

Recruitment

Patients were recruited on the basis of SARS-CoV-1 infection, BA.1 infection and SARS-CoV-2 vaccination. The only exclusion criteria used were HIV or other debilitating diseases. All SARS convalescents were infected by SARS-CoV-1 in 2003; thus, the average age of this cohort is older than other cohorts involved in this study. This may cause a systematically lower humoral immunity response in the SARS convalescents cohort.

Ethics oversight

This study was approved by the Ethics Committee of Beijing Ditan Hospital affiliated to Capital Medical University (Ethics committee archiving No. LL-2021-024-02), the Tianjin Municipal Health Commission, and the Ethics Committee of Tianjin First Central Hospital (Ethics committee archiving No. 2022N045KY). Written informed consent was obtained from each participant in accordance with the Declaration of Helsinki. All participants provided written informed consent for the collection of information, and that their clinical samples were stored and used for research. Data generated from the research were agreed to be published.

Note that full information on the approval of the study protocol must also be provided in the manuscript.

Flow Cytometry

Plots

Confirm that:

☒ The axis labels state the marker and fluorochrome used (e.g. CD4-FITC).

☒ The axis scales are clearly visible. Include numbers along axes only for bottom left plot of group (a 'group' is an analysis of identical markers).

☒ All plots are contour plots with outliers or pseudocolor plots.

☒ A numerical value for number of cells or percentage (with statistics) is provided.

Methodology

Sample preparation

Whole blood samples from SARS-CoV-2, SARS convalescents or vaccinees were mixed and subjected to Ficoll (Cytiva, 17-1440-03) gradient centrifugation after 1:1 dilution in PBS+2% FBS. After centrifugation, plasma was collected from upper layer and cells were harvested at the interface, respectively. PBMCs were further prepared through centrifugation, red blood cells lysis (InvitrogenTM eBioscienceTM 1X RBC Lysis Buffer, 00-4333-57) and washing steps. Samples were stored in FBS (Gibco) with 10% DMSO (Sigma) in liquid nitrogen if not used for downstream process immediately. Cryopreserved PBMCs were thawed in DPBS+2% FBS (Stemcell, 07905). On the day of sorting, B cells were enriched using CD19+ B cell isolation kit according to the manufacturer's instructions (STEMCELL, 19054). Enriched B cells were then stained with FITC anti-human CD20 antibody, Brilliant Violet 421™ anti-human CD27 antibody, PE/Cyanine7 anti-human IgM antibody, PE/Cyanine7 anti-human IgD antibody(BioLegend, 348210), biotinylated SARS-CoV-2 BA.1 protein (His & AVI Tag) (SinoBiological, 40592-V49H7-B) conjugated with PE-streptavidin, APC-streptavidin, TotalSeq™-C0971 Streptavidin (BioLegend, 405271), and TotalSeq™-C0972 Streptavidin (BioLegend, 405273), SARS-CoV-2 biotinylated RBD protein (His & AVI Tag) conjugated with Brilliant Violet 605™ Streptavidin, TotalSeq™-C0973 Streptavidin(BioLegend, 405275), TotalSeq™-C0974 Streptavidin(BioLegend, 405277), biotinylated Ovalbumin conjugated with TotalSeq™-C0975 Streptavidin(BioLegend, 405279) and 7-AAD.

Instrument

Astrios EQ (BeckMan Coulter)

Software	Summit 6.0 (Beckman Coulter) for cell sorting; FlowJo 10.8 for data analysis.
Cell population abundance	Memory B cell purity post-sorting is over 90% as measured by 10x sequencing.
Gating strategy	7-AAD-, CD20+, CD27+, IgM-, IgD-, SARS-CoV-2 BA.1 RBD+ B cells were sorted on an Astrios EQ (BeckMan Coulter) into PBS containing 30% FBS. The detailed FSC/SSC gating scheme is showed in Extended Data Figure 2. Gates are drown to define positive cells on the basis of unvaccinated healthy donor control.

☒ Tick this box to confirm that a figure exemplifying the gating strategy is provided in the Supplementary Information.
